# Traditional and HIV-specific risk factors for cardiovascular morbidity and mortality among HIV-infected adults in Brazil: a retrospective cohort study

**DOI:** 10.1186/s12879-016-1735-4

**Published:** 2016-08-08

**Authors:** Chanelle M. Diaz, Eddy R. Segura, Paula M. Luz, Jesse L. Clark, Sayonara R. Ribeiro, Raquel De Boni, Leonardo Eksterman, Rodrigo Moreira, Judith S. Currier, Valdiléa G. Veloso, Beatriz Grinsztejn, Jordan E. Lake

**Affiliations:** 1 UCLA David Geffen School of Medicine, University of California, 11075 Santa Monica Blvd. St. 100, Los Angeles, 90025 CA USA; 2 Montefiore University Hospital of Albert Einstein College of Medicine, Bronx, NY USA; 3 Fundação Oswaldo Cruz, Instituto Nacional de Infectologia Evandro Chagas, Rio de Janeiro, Brazil

**Keywords:** HIV, AIDS, Cardiovascular disease, Antiretroviral therapy, Brazil

## Abstract

**Background:**

Antiretroviral therapy (ART) agents potentially associated with adverse metabolic profiles are commonly used in low- and middle-income countries. We assessed risk factors for cardiovascular disease (CVD)-related morbidity and mortality in a cohort of HIV-infected, ART-treated adults in Rio de Janeiro, Brazil.

**Methods:**

Hospital records and mortality data between 2000–2010 were examined for incident CVD-related ICD-10 and Coding of Death in HIV diagnoses among adults ≥18 years old on ART, enrolled in an observational cohort. Poisson regression models assessed associations between demographic and clinical characteristics and ART agent or class on CVD event risk.

**Results:**

Of 2960 eligible persons, 109 had a CVD event (89 hospitalizations, 20 deaths). Participants were 65 % male, 54 % white, and had median age of 37 and 4.6 years on ART. The median nadir CD4^+^ T lymphocyte count was 149 cells/mm^3^. The virologic suppression rate at the end of study follow-up was 60 %. In multivariable models, detectable HIV-1 RNA prior to the event, prior CVD, less time on ART, age ≥40 at study baseline, nadir CD4^+^ T lymphocyte count ≤50 cells/mm^3^, non-white race, male gender, and a history of hypertension were significantly associated with CVD event incidence (*p* < 0.05), in order of decreasing strength. In multivariate models, cumulative use of tenofovir, zidovudine, efavirenz and ritonavir-boosted atazanavir, darunavir and/or lopinavir were associated with decreased CVD event risk. Recent tenofovir and boosted atazanavir use were associated with decreased risk, while recent stavudine, nevirapine and unboosted nelfinavir and/or indinavir use were associated with increased CVD event risk.

**Conclusions:**

Virologic suppression and preservation of CD4^+^ T-lymphocyte counts were as important as traditional CVD risk factor burden in determining incident CVD event risk, emphasizing the overall benefit of ART on CVD risk and the need for metabolically-neutral first- and second-line ART in resource-limited settings.

**Electronic supplementary material:**

The online version of this article (doi:10.1186/s12879-016-1735-4) contains supplementary material, which is available to authorized users.

## Background

With successful antiretroviral therapy (ART), HIV infection has been transformed into a chronic disease [1]. Worldwide and in Brazil, AIDS-related morbidity and mortality has declined, whereas the burden of non-communicable diseases, such as cardiovascular disease (CVD), has increased [2–6]. Specifically, CVD risk is up to two-fold higher among HIV-infected persons compared with HIV-uninfected individuals, even after adjusting for traditional CVD risk factors [7–9]. And, while higher frequencies of traditional CVD risk factors have been reported in HIV-infected persons, the impact of HIV on CVD is most prominent in traditionally low-risk demographic groups [10–13], suggesting CVD risk in treated HIV infection may also be mediated by non-traditional factors.

In Brazil, CVD incidence has risen more rapidly among HIV-infected persons than the general population [14], and hospitalization costs for myocardial infarctions have surpassed those for AIDS-related illnesses [15]. However, the dual burden of CVD and HIV in low- and middle-income countries is understudied [16, 17]. Discerning modifiable CVD risk factors in treated HIV infection may help prevent and treat CVD, reducing disease burden and health system costs while improving quality of life.

While ART is critical to successful control of HIV infection, several observational studies have reported associations between specific antiretroviral agents and increased CVD risk [18–24]. Data are conflicting, however, and the evidence is predominantly derived from U.S. and European cohorts, whose relevance to low- and middle-income countries that routinely use ART with poorer metabolic profiles remains unclear. Understanding whether commonly used ART regimens may be contributing to CVD risk is a critical component of long-term HIV management that may both inform global ART guidelines and improve clinical practice in resource-limited settings. This analysis aims to define traditional and HIV-specific risk factors for CVD in a large cohort of HIV-infected adults on ART in Rio de Janeiro, Brazil, including whether specific antiretroviral agents and classes confer additional risk of CVD morbidity and mortality.

## Methods

### Study setting and population

**Fig. 1:**
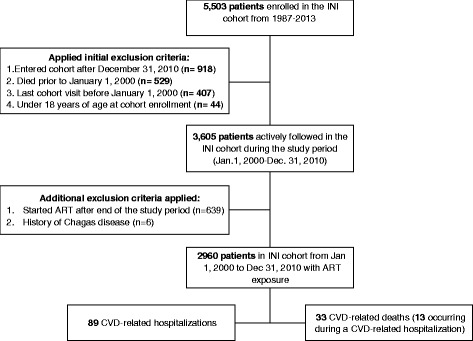
Study Participant Flowchart

The Instituto Nacional de Infectologia Evandro Chagas (INI) of Fundação Oswaldo Cruz (FIOCRUZ) in Rio de Janeiro, Brazil is a national public referral center for HIV/AIDS care. Since 1998, INI has maintained an observational, longitudinal, open cohort of adults receiving HIV primary care at their clinic. Complete cohort procedures have previously been described [25]. Trained abstractors update the clinical database biannually using outpatient and inpatient medical records, laboratory results, and ART usage data. This analysis included all HIV-infected persons ≥18 years of age with ART exposure (and without a history of Chagas disease) who were followed in the INI cohort between January 1, 2000 and December 31, 2010 (Fig. 1).

### Study design and outcomes

The primary outcome was a composite endpoint that included both hospitalizations and deaths related to CVD, whichever occurred first. Secondary outcomes included separate analyses of hospitalization and death outcomes. Participants were censored after their first hospitalization, at the time of death, or at end of the study period, whichever came first. For both hospitalizations and deaths, CVD-related events included heart or vascular disease, ischemic heart disease, stroke, venous thromboembolism and pulmonary embolism.

Hospitalizations for CVD-related illnesses were identified using physician-reported discharge diagnoses linked to an *International Classification of Diseases*, *Tenth Revision* (ICD-10) code. Clinical diagnoses for cohort participants hospitalized at INI were retrieved and systematically checked and validated by two clinicians with experience in the management of HIV infection. All diagnoses were classified using ICD-10 codes and then allocated into 24 categories. From this list, only CVD-related diagnoses were included in this analysis. For hospitalizations with multiple CVD diagnoses, the primary cause was determined using a hierarchical approach that prioritized acute over chronic disease. A complete list of ICD-10 codes included in this analysis is provided in Table 1. Of note, 10 % of all hospitalization data (CVD and non-CVD) were randomly selected for discharge diagnosis adjudication by a non-treating physician specializing in HIV care as a quality control measure. Diagnosis validation required an objective medical test or other document, such as a consultation note, discharge summary, or autopsy report.

**Table 1:** Distribution of ICD-10 hospital diagnoses and CoDe causes of death

ICD-10 Code	CVD-Related Hospital Discharge Diagnoses	*N* = 89, *n* (%)
I82.9	Venous embolism and thrombosis	27 (30.3)
I10 & I11	Hypertension	17 (19.1)
I50.0	Congestive heart failure	12 (13.5)
I63.9	Cerebral infarction	10 (11.2)
I20.9	Angina pectoris	6 (6.7)
I21.9	Acute myocardial infarction	6 (6.7)
J81.0	Pulmonary edema	4 (4.5)
I61.9	Intracerebral hemorrhage	2 (2.3)
I24.8	Acute Ischemic heart disease	1 (1.1)
I26.9	Pulmonary embolism	1 (1.1)
I42.9	Cardiomyopathy	1 (1.1)
I50.1	Left ventricular failure	1 (1.1)
I81.0	Portal vein thrombosis	1 (1.1)
CoDe No.	CVD-Related Causes of Death	*N* = 33, *n* (%)
24	Heart or vascular disease	18 (54.4)
8	Ischemic heart disease	10 (30.3)
9	Stroke	4 (12.1)
12	Lung embolus	1 (3.0)

For deaths, the “Coding of Death in HIV” (CoDe) method was used to determine cause of death [3]. This method requires detailed data collection on the causes of death and contributing factors by a physician specializing in HIV care, applies a uniform coding system and includes an independent centralized review process performed by two additional HIV specialists [26]. Information regarding vital statistics was exhaustively checked up until December 31, 2010 using patients’ INI medical charts, through active contact with individuals and family members, and by linkage with the Rio de Janeiro State mortality database using a previously validated algorithm [3, 27]. Only primary, CVD-related causes of death were included (Table 1), with the exception of six cases where CVD was determined to be a major contributing factor.

Cumulative and recent exposure to specific antiretroviral drugs and classes were calculated [see Additional file 1 for the complete cohort ART exposure history]. Nucleoside reverse transcriptase inhibitor (NRTI), non-NRTI (NNRTI), protease inhibitor (PI), and integrase inhibitor classes were included. No minimum exposure was required. The cumulative exposure is reported as the summation of an individual’s time on a given agent or drug class, up to the end of their study follow-up. Recent use was defined as any exposure in the 6 months preceding the event or end of follow-up.

Age at study start was calculated as the difference between date of birth and the start of the study period. Race/ethnicity was based on provider report and was dichotomized as white or non-white. Education was self-reported and dichotomized as either eight or fewer years of schooling or greater than 8 years. Presumed HIV exposure from injection drug use was self-reported, whereas cocaine use was provider reported. Time since HIV diagnosis was calculated as the difference between the self-reported or laboratory documented date of HIV diagnosis and the study start date. Percent time with known HIV on ART was calculated by dividing cumulative ART exposure by the difference between the date of HIV diagnosis and the end of study follow-up. Nadir CD4^+^ T lymphocyte count was defined as the lowest recorded measurement obtained at least 7 days prior to the end of follow-up, either on or off of ART, and was categorized as ≤ 50 cells/mm^3^ or greater. To account for changing sensitivities of HIV-1 RNA assays over the study period, virologic suppression was defined as ≤ 400 copies/mL. Viremia copy-years were estimated to quantify cumulative viremia, defined as the number of HIV-1 RNA copies per mL per year, integrated over the number of years since HIV seroconversion [28].

Traditional CVD risk factors were evaluated until the end of study follow-up and included diabetes mellitus, hypertension, dyslipidemia, prior CVD, smoking, chronic kidney disease, and weight. Diabetes mellitus was defined as either prior diagnosis of diabetes mellitus on treatment, two fasting glucose level ≥126 mg/dL without symptoms, one fasting glucose level ≥126 mg/dL with symptoms, or a hemoglobin A1c value of 6.5 or greater. Dyslipidemia was defined by use of lipid-lowering therapy, low-density lipoprotein (LDL) cholesterol >159 mg/dL, high-density lipoprotein (HDL) cholesterol <40 mg/dL, total cholesterol >239 mg/dL, or triglycerides >199 mg/dL. Similarly, hypertension was defined as prior diagnosis of hypertension, use of antihypertensive medication, systolic blood pressure >140 mmHg, or diastolic blood pressure >90 mmHg. Prior CVD included any history of stable or unstable angina, cerebrovascular disease, or myocardial infarction per the medical record. Smoking history was comprised of a cross-sectional survey of smoking and self-reported nicotine use. Each participant was categorized as ever smoker, never smoker, or unknown. Upon further analysis, persons with an unknown smoking history (*n* = 534, 18 %) were found to have a significantly increased risk of CVD-related events (data not shown), and were thus reclassified as ever-smokers to account for any possibility that smoking is causing the outcome of study rather than ART exposure. History of chronic kidney disease was defined as provider report or end-stage renal disease requiring dialysis. A significant proportion of patients were missing height data for body mass index calculation/obesity assessment. As such, weight was used as a surrogate measure for obesity, with the highest weight prior to the end of follow-up used to create weight tertiles.

### Statistical analysis

Study entry was defined as either January 1, 2000 or the date of cohort enrollment (for participants enrolling after January 1, 2000). End of follow-up was defined as the first occurrence of a CVD-related hospitalization, death due to any cause, or December 31, 2010 (except when death was analyzed separately and participants were not censored after hospitalizations). Mortality data were also gathered from public records.

Descriptive statistics for demographic and clinical variables were compared using Chi-squared and Wilcoxon rank sum tests for categorical and continuous variables, respectively (two-sided α = 0.05). Poisson regression models using individual-level data explored associations between traditional and HIV-specific CVD risk factors (including ART exposure) and risk of cardiovascular morbidity and/or mortality. Incidence rate ratios for CVD events were calculated, with person-time defined as the sum of an individual’s years of study follow-up incorporated as an offset. We used a two-step approach to our multivariate modeling: We initially adjusted for age; sex; race; education; nadir CD4^+^ T lymphocyte count; percent time with known HIV on ART; virologic suppression in the year prior to the event; history of diabetes mellitus, hypertension, dyslipidemia, chronic kidney disease, or prior CVD; smoking history; weight; and calendar year of cohort entry. Covariates with *p* < 0.10 were retained [see Additional file 2, which details the process of arriving at the final multivariate regression model]. ART classes and agents were then added individually to the model to estimate additional CVD risk attributable to ART. Adding ART exposure to the model obligated exclusion of the percent time with known HIV on ART variable due to colinearity. Participants not on ART in the 6 months prior to an event were excluded from the analysis of recent ART exposure.

For the composite endpoint, the model included: age ≥40 years; male sex; non-white race; nadir CD4^+^ T lymphocyte count ≤50cells/mm^3^; percent time with known HIV on ART; virologic suppression; history of hypertension, dyslipidemia, or CVD; and calendar year of cohort entry. Briefly, for hospitalizations, the model was similar to the composite endpoint model except that history of diabetes mellitus but not prior CVD remained significant. For deaths, the model included age ≥40, ≤ 8 years of schooling, virologic suppression in the last year, and ≥ 1 metabolic risk factor (diabetes mellitus, hypertension, untreated dyslipidemia, or chronic kidney disease).

Missing nadir CD4^+^ T lymphocyte count (*n* = 67, 2 %) and HIV-1 RNA (*n* = 448, 15 %) data were generated using multiple imputation, with age, sex, and the percent time with known HIV on ART as predictors. Missing weight (*n* = 91, 3 %) was replaced by median, sex-specific cohort weight. Missing race (*n* = 9, 0.3 %) and education (*n* = 13, 0.4 %) were replaced with the highest risk category. Bivariate Poisson regression models demonstrated similar results for multiple imputation versus exclusion of missing data. Multiple testing was corrected using the Benjamini-Hochberg method [29, 30]. Stata (version 12.0, StataCorp, College Station, TX) was used for all analyses.

## Results

### Cohort characteristics

INI cohort participants (*n* = 2960) contributed 16,140 person-years (PY) of follow-up. The median follow-up was 4.68 years [interquartile range (IQR), 2.34–9.09]). Baseline characteristics included: median age of 37 years (IQR, 30, 43), 65 % male, 54 % white, and 53 % had eight or fewer years of schooling (Table 2). Two percent of participants reported a history of injection drug use and 9 % reported cocaine use. The median nadir CD4^+^ T lymphocyte count was 149 cells/mm^3^. Median time on ART was 4.65 years, with 95 % of participants on ART in the 6 months prior to their event/end of follow-up. Sixty percent of participants had undetectable HIV-1 RNA in the year prior to their event/end of follow-up.

**Table 2:** Characteristics and Cardiovascular Risk Profile of INI Cohort Participants Exposed to ART from 2000–2010

				CVD Events			VTE events
		All study participants	No CVD-related events	Composite CVD events†	CVD-related hospitalization	CVD-related death	VTE hospitalizations and death
		*n*	*n* (%)	*n* (%)	*n* (%)	*n* (%)	*N* (%)
Demographic & clinical characteristics		2,960	2,851 (96 %)	109 (4 %)	89 (3 %)	33 (1 %)	30 (1 %)
Median Age at study start (IQR)		37 (30, 43)	36 (30, 43)	42 (35, 51)*	40 (35, 50)*	46 (37, 58)*	36.5 (31, 44)
Age	<30 years	710 (24 %)	692 (24 %)	18 (17 %)	14 (16 %)	5 (15 %)	7 (23 %)
	30–39 years	1,107 (37 %)	1,080 (38 %)	27 (25 %)	25 (28 %)	6 (18 %)**	11 (37 %)
	≥40 years	1,143 (39 %)	1,079 (25 %)	64 (59 %)	50 (56 %)	22 (67 %)	12 (40 %)
Male Gender		1,924 (65 %)	1,849 (65 %)	75 (69 %)	64 (72 %)	21 (64 %)	23 (77 %)
White Race		1,605 (54 %)	1,554 (55 %)	51 (47 %)	40 (45 %)	15 (45 %)	12 (40 %)
≤8 years of Education		1,556 (53 %)	1,487 (52 %)	69 (64 %)**	57 (64 %)**	24 (73 %)**	16 (53 %)
History of IDU		61 (2 %)	58 (2 %)	3 (3 %)	3 (3 %)	1 (3 %)	0 (0 %)
History of Cocaine Use		262 (9 %)	255 (9 %)	7 (6 %)	6 (7 %)	1 (3 %)	2 (7 %)
Time since HIV diagnosis, Median years (IQR)		1.12 (0.13, 5.78)	1.08 (0.13, 5.7)	3.06 (0.24, 6.72)*	3.06 (0.29, 6.5)*	4.7 (0.24, 7.56)	1.35 (0.13, 3.22)
% of time on ART since HIV diagnosis		0.77 (0.47, 0.93)	0.77 (0.47, 0.93)	0.72 (0.43, 0.89)	0.71 (0.42, 0.89)	0.87 (0.52, 0.98)	0.67 (0.41, 0.93)
Median Nadir CD4+ T-cell count, cells/mm^3^ (IQR)		149 (52, 258)	152 (53, 259)	97 (27, 189)*	88.5 (23, 203)*	104.5 (23.5, 188.5)*	91 (27.5, 182.5)*
Nadir CD4+ T-cell count ≤ 50 cecells/mm^3^		705 (24 %)	666 (23 %)	39 (36 %)**	33 (37 %)**	13 (39 %)**	11 (37 %)
Virally suppressed year before event		1,771 (60 %)	1,734 (61 %)	37 (34 %)**	29 (33 %)**	14 (42 %)**	9 (30 %)**
Median viremia copy-years, log_10_ copy x years/mL (IQR)		4.72 (3.99, 5.34)	4.71 (3.98, 5.34)	4.94 (4.33, 5.67)	4.93 (4.28, 5.66)*	4.43 (3.76, 5.06)	5.08 (4.73, 5.82)*
Cardiovascular risk factors							
History of Diabetes Mellitus		320 (11 %)	299 (10 %)	21 (19 %)**	18 (20 %)**	5 (15 %)	5 (17 %)
History of Hypertension		792 (27 %)	740 (26 %)	52 (48 %)**	42 (47 %)**	19 (58 %)**	9 (30 %)
History of Dyslipidemia^a^		1,487 (50 %)	1,429 (50 %)	58 (53 %)	44 (49 %)	21 (64 %)	11 (37 %)
History of Chronic kidney disease		25 (<1 %)	23 (<1 %)	2 (2 %)	2 (2 %)	1 (3 %)	0 (0 %)
>1 metabolic risk factors for CVD		1,813 (61 %)	1,734 (61 %)	79 (72 %)**	62 (70 %)	29 (88 %)**	17 (57 %)
Prior CVD^b^		97 (3 %)	81 (3 %)	16 (15 %)**	9 (10 %)**	11 (33 %)**	2 (7 %)
Ever smoked		2,013 (68 %)	1,926 (68 %)	87 (80 %)**	67 (75 %)	33 (100 %)**	21 (70 %)
Median weight kg (IQR)~		71.3 (62.5, 81.3)	71.6 (62.8, 81.4)	67.6 (60.5, 78.5)	67.5 (60.1, 78.5)	68.6 (60.3, 88)	68.8 (61, 79.2)

*Abbreviations*: *IQR* interquartile range, *CVD* cardiovascular disease

†Composite CVD events include all CVD-related hospitalizations and deaths listed in Table 1

*Rank sum test of comparison of event and non-event groups *p* < 0.05

**Chi square test of comparison of event and non-event groups *p* < 0.05

~based on the highest weight recorded prior to the end of follow-up or time of event

^a^includes any lab confirmed history of dyslipidemia, hypercholesterolemia, or hypertriglyceridemia, and patients on lipid-lowering drugs

^b^Prior CVD = history of MI, CAD, or stroke

CVD risk factors were highly prevalent, particularly among participants experiencing CVD events, with 61 % reporting one or more metabolic risk factors (diabetes mellitus, hypertension, dyslipidemia, and/or chronic kidney disease). Smoking was the most common risk factor (68 %), followed by dyslipidemia (50 %), and hypertension (27 %), with prior CVD present in 3 % of participants. Forty-three percent of participants with a history of dyslipidemia had a documented treatment history, while 72 % of participants with hypertension received treatment. Of the available body mass index (BMI) data, only 13 % had a BMI over 30.

### Cardiovascular events

During the study period, 109 persons had a CVD-related event (89 hospitalizations; 33 deaths, 13 of which occurred during hospitalization, Table 2), for an overall incidence rate of 6.75 per 1000 PY (95 % CI: 5.60–8.15). Thirteen deaths were excluded from the composite endpoint because the death occurred after a hospitalization/censoring. Hospitalization and death incidence rates were 5.51 per 1000 PY (95 % CI: 4.48–6.79) and 2.02 per 1000 PY (95 % CI: 1.43–2.84), respectively. The incidence rates for thrombotic events and ischemic heart disease were 4.71 per 1000 PY (95 % CI: 3.76–5.90) and 1.80 per 1000 PY (95 % CI: 1.25–2.59), respectively.

**Fig. 2:**
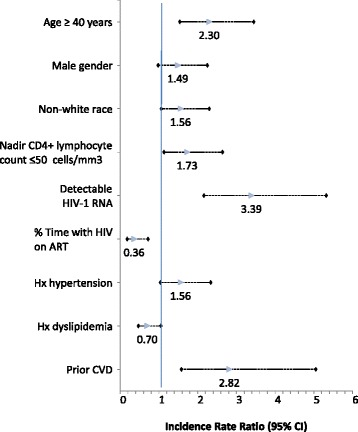
Adjusted* incidence rate ratios for associations between cardiovascular disease (CVD) events and patient demographics and clinical characteristics. *Adjusted for all covariates listed

In unadjusted analyses, age ≥40 years, non-white race, 8 years or fewer of schooling, nadir CD4^+^T lymphocyte count ≤50 cells/mm^3^, detectable HIV-1 RNA, smoking, a history of diabetes mellitus, hypertension, or CVD and weight in the lowest tertile were associated with incident CVD [see Additional file 3, which details the crude incidence rate ratios for demographic/clinical characteristics and cardiovascular risk factors]. Longer time on ART after HIV diagnosis was significantly protective. In multivariate analysis, age ≥40 years, non-white race, nadir CD4^+^T lymphocyte count ≤50 cells/mm^3^, detectable HIV-1 RNA, and a history of hypertension or CVD remained significant (Fig. 2). Detectable HIV-1 RNA in the prior year (crude incidence rate ratio (cIRR), 3.99; 95 % CI, 2.61–6.11) and prior CVD (cIRR, 3.99; 95 % CI, 2.35–6.79) were the factors most strongly associated with a CVD event.

### ART exposure

**Fig. 3:**
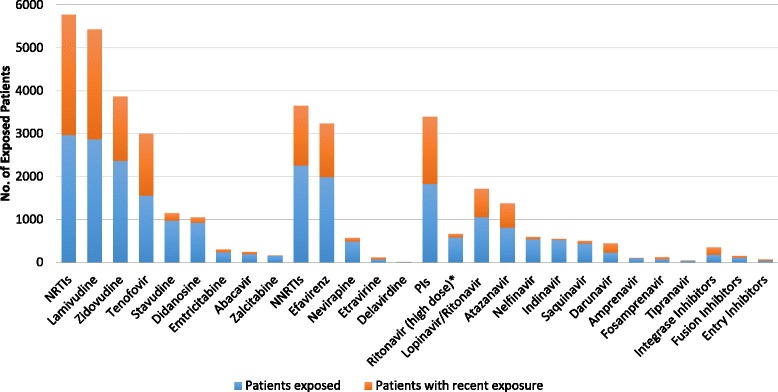
INI Cohort ART Treatment History

Overall, the cohort had 18,127 PY of cumulative ART exposure, Fig. 3 maps out the Cohort ART treatment history. All participants were NRTI-exposed (18,116 PY), 76 % had NNRTI exposure (7411 PY), and 62 % had PI exposure (9973 PY). Integrase, fusion, and entry inhibitors were used by a minority of participants (6, 4, and 1 %, respectively). The most commonly used agents were lamivudine (97 %), zidovudine (80 %), tenofovir (53 %), stavudine (33 %), didanosine (31 %), efavirenz (67 %), ritonavir-boosted lopinavir (36 %), and ritonavir-boosted atazanavir (21 %), in keeping with Brazilian treatment guidelines during the follow-up period.

### Impact of ART exposure

After adjusting for traditional and HIV-specific CVD risk factors, cumulative NRTI exposure was associated with a 21 % lower CVD event incidence (adjusted IRR (aIRR), 0.79; 95 % CI, 0.75–0.83, Table 3). Specifically, cumulative lamivudine, zidovudine, and tenofovir exposure were associated with decreased CVD event incidence. Cumulative tenofovir exposure had the strongest association, with a 40 % decrease in CVD event incidence per additional year of exposure (aIRR, 0.60; 95 % CI, 0.51–0.71). Recent stavudine use was associated with a 528 % increase in CVD event incidence (aIRR, 5.28; 95 % CI 3.18–8.75), whereas recent tenofovir exposure was associated with a 63 % decreased CVD event incidence (aIRR, 0.37; 95 % CI, 0.24–0.56).

**Table 3:** Crude and Adjusted ^a^ Incidence Rate Ratios for CVD Events by Exposure to Specific Antiretroviral Drugs

		Cumulative exposure						Recent exposure					
	*N*	cIRR	95 % CI	*P*	aIRR	95 % CI	*P*	cIRR	95 % CI	*P*	aIRR	95 % CI	*P*
ART	109	0.85	0.81–0.89	<0.001	0.79	0.75–0.83	<0.001	–	–	–	–	–	–
NRTI	109	0.85	0.81–0.89	<0.001	0.79	0.75–0.83	<0.001	–	–	–	–	–	–
Lamivudine	105	0.80	0.75–0.84	<0.001	0.76	0.72–0.81	<0.001	0.56	0.30–1.05	0.069	0.71	0.37–1.36	0.31
Zidovudine	93	0.86	0.81–0.91	<0.001	0.83	0.78–0.89	<0.001	1.27	0.86–1.89	0.23	1.34	0.90–2.00	0.15
Tenofovir	39	0.64	0.55–0.75	<0.001	0.60	0.51–0.71	<0.001	0.42	0.28–0.64	<0.001	0.37	0.24–0.56	<0.001
Stavudine	56	0.97	0.90–1.05	0.50	0.96	0.88–1.04	0.30	6.13	3.91–9.60	<0.001	5.28	3.18–8.75	<0.001
Didanosine	51	0.95	0.87–1.04	0.29	0.91	0.82–1.02	0.10	1.47	0.68–3.17	0.32	1.50	0.68–3.27	0.32
Emtricitabine	5	0.78	0.49–1.25	0.30	0.83	0.51–1.36	0.46	2.29	0.73–7.23	0.16	2.61	0.81–8.43	0.11
Abacavir	6	0.70	0.43–1.14	0.15	0.63	0.36–1.11	0.11	0.97	0.24–3.94	0.97	0.70	0.17–2.86	0.62
Zalcitabine	10	1.01	0.66–1.55	0.96	0.90	0.57–1.41	0.64	–	–	–	–	–	–
NNRTI	79	0.76	0.69–0.83	<0.001	0.77	0.70–0.85	<0.001	1.05	0.72–1.55	0.79	1.42	0.94–2.13	0.097
Efavirenz	67	0.75	0.67–0.84	<0.001	0.78	0.69–0.87	<0.001	0.90	0.60–1.34	0.59	1.25	0.81–1.93	0.31
Nevirapine	31	0.92	0.81–1.05	0.24	0.91	0.78–1.05	0.20	2.46	1.20–5.06	0.014	3.07	1.44–6.53	0.004
PI	79	0.92	0.88–0.96	0.001	0.88	0.83–0.93	<0.001	1.20	0.80–1.81	0.37	0.79	0.50–1.23	0.29
PI (without high dose RTV)	78	0.92	0.87–0.96	<0.001	0.87	0.83–0.92	<0.001	1.16	0.77–1.73	0.48	0.75	0.48–1.17	0.20
Ritonavir	35	0.91	0.78–1.05	0.20	0.84	0.71–1.01	0.061	4.05	2.05–8.03	<0.001	2.67	1.30–5.47	0.007
Lopinavir/ Ritonavir	41	0.86	0.76–0.96	0.009	0.81	0.71–0.92	0.001	1.20	0.79–1.84	0.40	0.90	0.58–1.39	0.64
Atazanavir + Ritonavir	13	0.60	0.45–0.79	<0.001	0.60	0.45–0.81	0.001	0.36	0.19–0.69	0.002	0.40	0.21–0.76	0.006
Darunavir + Ritonavir	6	0.71	0.50–1.02	0.067	0.65	0.45–0.95	0.027	0.64	0.28–1.46	0.29	0.47	0.20–1.09	0.08
Nelfinavir without booster	28	0.97	0.87–1.09	0.66	0.96	0.84–1.09	0.50	7.77	4.05–14.91	<0.001	5.32	2.60–10.88	<0.001
Indinavir without booster	39	1.05	0.95–1.16	0.35	1.00	0.89–1.11	0.98	11.56	5.84–22.90	<0.001	10.22	4.63–22.53	<0.001
Saquinavir + Ritonavir	4	0.55	0.15–2.0	0.37	0.47	0.11–2.12	0.33	1.63	0.23–11.68	0.63	1.05	0.14–7.58	0.97
Saquinavir without booster	24	0.90	0.71–1.13	0.35	0.82	0.62–1.08	0.15	3.96	1.46–10.76	0.007	2.07	0.71–6.00	0.18
Amprenavir + Ritonavir	3	1.55	0.82–2.93	0.18	1.37	0.66–2.85	0.39	–	–	–	–	–	–
Amprenavir without booster	4	0.85	0.42–1.73	0.66	0.64	0.28–1.49	0.30	9.62	1.34–68.95	0.024	3.88	0.52–28.89	0.19
Integrase Inhibitor	4	0.82	0.53–1.28	0.39	0.78	0.50–1.22	0.28	0.62	0.23–1.69	0.35	0.54	0.20–1.49	0.24

*Abbreviations*: *CVD* cardiovascular disease, *N* number of participants with a CVD-related event exposed to the specific agent, *cIRR* crude incidence rate ratio, *CI* confidence interval, *P p*-value, *ART* combined antiretroviral therapy, *NRTI* nucleoside reverse transcriptase inhibitor, *NNRTI* non-NRTI, *PI* protease inhibitor, *RTV* ritonavir

^a^Final model was adjusted for specific ART agent or class; age ≥40 years; male sex; non-white race; nadir CD4^+^ T lymphocyte count ≤50 cells/mm^3^; virologic suppression; history of hypertension, dyslipidemia, or CVD; and calendar year of cohort entry

Cumulative NNRTI exposure was significantly associated with decreased CVD event incidence (aIRR, 0.77; 95 % CI, 0.70–0.85, Table 3), an association driven by efavirenz use (25 % lower event incidence; aIRR, 0.78; 95 % CI, 0.69–0.87). No effect of cumulative nevirapine exposure was observed; however, recent nevirapine exposure was associated with an overall increased CVD event incidence (aIRR, 3.07; 95 % CI, 1.44–6.53). Neither recent NNRTI class nor recent efavirenz exposure were significantly associated with CVD event incidence.

Cumulative PI exposure was associated with a 12 % decreased CVD event incidence (aIRR 0.88; 95 % CI, 0.83–0.93). This effect did not change significantly when high-dose ritonavir exposure was excluded (Table 3). Each additional year of exposure to ritonavir-boosted lopinavir (aIRR, 0.81; 95 % CI, 0.71–0.92), atazanavir (aIRR, 0.60; 95 % CI, 0.45–0.81), and darunavir (aIRR, 0.65; 95 % CI, 0.45–0.95) was associated with decreased CVD event incidence (19, 40, and 35 %, respectively). Recent ritonavir-boosted atazanavir exposure was associated with a 60 % decreased CVD event incidence (aIRR, 0.40; 95 % CI, 0.21–0.76), whereas recent high-dose ritonavir (aIRR, 2.67; 95 % CI, 1.30–5.47), unboosted nelfinavir (aIRR, 5.32; 95 % CI, 2.60–10.88) and unboosted indinavir (aIRR, 10.22; 95 % CI, 4.63–22.53) exposure were associated with increased CVD event incidence.

When hospitalizations and deaths were analyzed separately, associations with ART use were similar (see Additional file 4, which demonstrate the crude and adjusted incidence rate ratios for cardiovascular hospitalizations and deaths by exposure to specific ART agents). Sensitivity analysis excluding venous thromboembolism and pulmonary embolism from the primary outcome variable did not significantly change results [see Additional file 5 for further discussion of and results from the sensitivity analysis]. Additionally, neither excluding participants with prior CVD nor restricting the PI exposure analysis to participants with PI and NNRTI exposure significantly altered results, although traditional CVD risk factors became more prominent when prior CVD was excluded (data not shown). After adjustment for multiple testing, cumulative darunavir use was the only result to lose significance.

## Discussion

We sought to clarify the link between traditional and HIV-specific risk factors and cardiovascular morbidity and mortality in a large, well-characterized cohort of HIV-infected adults on ART in a middle-income country. We observed a significant burden of traditional CVD risk factors compared with the general Brazilian population [31]. Notably, our cohort was young relative to previously-studied cohorts, with 61 % under 40 years of age [22, 23]. Similarly, although age 40 and over was associated with increased CVD event risk in multivariate analysis, 41 % of events occurred in persons less than 40 years of age (IR, 4.46 per 1000 PY; 95 % CI, 3.33–5.98). Additionally, 61 % of participants had at least one traditional CVD risk factor. While these rates are comparable to other studies of HIV-infected persons in Brazil and Latin America [32, 33], the risk factor burden for age is greater than previously observed CVD risk factor frequencies in the United States and Europe [22, 24, 34, 35].

HIV-specific risk factors were more strongly associated with incident CVD morbidity and mortality than traditional CVD risk factors, including nadir CD4^+^ T lymphocyte count ≤50 cells/mm^3^ and detectable HIV-1 RNA in the year prior to the event. These factors remained independently associated with incident CVD after controlling for time on ART, consistent with literature citing low nadir CD4^+^ T lymphocyte count and ongoing HIV replication as risk factors for CVD [36–39], but countering the belief that traditional CVD risk factors account for the majority of CVD risk in the setting of HIV infection [40]. Even though ART is universally available through Brazil’s public health system at no cost, late diagnosis remains a problem, as evidenced by low nadir CD4^+^ T lymphocyte counts [41]. Moreover, virologic suppression rates were sub-optimal at 60 %, and poorer adherence may be related to the toxicity of the available first and second line regimens [42].

Low nadir CD4^+^ T lymphocyte count and ongoing HIV replication are linked to chronic immune activation and inflammation in HIV-infected persons, contributing to atherosclerosis development via HIV-mediated endothelial injury and the promotion of a pro-thrombotic state [43]. Even though venous thromboembolism may traditionally be viewed as a distinct pathophysiologic entity from atherosclerotic CVD, there is an increasing body of evidence suggesting that patients with unprovoked venous thromboembolism are at a higher risk of arterial cardiovascular events than matched controls, and that venous and arterial thrombosis may instead be “two aspects of the same disease” [44–47]. The seemingly bi-directional relationship between coagulation and inflammation becomes significant in HIV infection since, in addition to a two-fold increased risk of myocardial infarction (MI), patients infected with HIV are at a two-to-ten-fold increased risk for a venous thromboembolic event [48–51]. Interestingly, levels of pro-thrombotic monocyte and tissue factor activation in HIV-infected persons with uncontrolled viremia mirror levels in HIV-uninfected persons experiencing acute coronary syndrome [52]. And, although some biomarkers of inflammation and immune activation decrease significantly with ART-induced virologic suppression, residual monocyte/macrophage activation persists and independently predicts mortality and disease burden in this population [53–59]. In light of the potential pathophysiologic link between these disease states, particularly in the chronic inflammatory state of HIV infection, the inclusion of thromboembolic events is a unique strength of this study.

We also examined associations between ART exposure during the study period and the risk of CVD events. Cumulative NRTI, NNRTI, and/or PI exposure were associated with a 12–23 % decreased CVD event incidence per additional year of exposure. Cumulative tenofovir and atazanavir use had the strongest associations, and these associations persisted both when hospitalizations and deaths were analyzed separately and when recent use was examined. In contrast, recent stavudine, nevirapine, high-dose ritonavir, unboosted nelfinavir, and unboosted indinavir exposure were associated with increased CVD event incidence. Neither cumulative nor recent abacavir exposure were significantly associated with CVD event incidence, although only 7 % of participants had abacavir exposure due to the high cost of HLA B5701 testing and the lack of generic formulations in Brazil. Our findings are in contrast to prior observational studies that detected an association between PI use and CVD [19, 23, 60–62]. Additionally, the D:A:D study group reported an increased risk of MI associated with cumulative indinavir and lopinavir-ritonavir exposure, as well as recent abacavir and didanosine exposure [20, 22]. Since then, results regarding CVD risk and abacavir and other ART exposure have been conflicting [24, 34, 35, 63, 64]. Interestingly, recently published data from the Swiss Cohort suggests that risk from recent abacavir exposure may confer cumulative risk, but only for a limited period, with exposure during the past six to 36 months causing the greatest increase in risk [65]. Coupled with their finding of a rapid early harmful effect for didanosine followed by a protective effect, these results suggest that there may be different processes mediating ART-associated CVD risk that are drug-specific [65].

This cohort differs from previously studied U.S. and European cohorts in that it is from a developing country setting with universal access to ART, but with a limited choice of agents that differs from what is commonly used in higher income settings. Until 2013, a zidovudine (AZT) backbone was the standard first line therapy, typically with lamivudine. Alternatives to AZT were didanosine or tenofovir. Abacavir was, and remains, rarely used due to its higher cost relative to other NRTIs, as is the case for emitricitabine. First line ART remains primarily NNRTI-based, with efavirenz being the preferred NNRTI, although PI use is allowed and commonly used as second line therapy. Lopinavir/ritonavir is the first choice PI, and atazanavir/ritonavir can be used as an optional PI. Third line and salvage therapy are also available and mostly based on agents such as darunavir, raltegravir, etravirine and enfuvirtide. The availability of such drugs has been expanded along the years, according to their availability in the market and their affordability in the Brazilian public health system. These results stress the importance of enhancing access to ART regimens with improved metabolic profiles as first and second line treatment in low- and middle-income settings, especially in the context of advanced HIV disease.

This study has several limitations, most significantly its observational design, which limited our ability to control for unknown sources of confounding and bias. Known sources of confounding were controlled for whenever possible, and treatment selection bias was minimized as physicians must prescribe in accordance with algorithmic Brazilian HIV treatment guidelines. We could not fully account for the effects of chronic kidney disease as time-updated glomerular filtration rates were not available. BMI was not available for all participants, and standardized screening was not present for family and social history variables, including substance use. Although we were not able to control for obesity due to missing BMI data, we observed a trend towards increased risk of CVD events with lower BMI (data not shown), and this trend remained consistent when weight tertiles were used. We were not able to control for concurrent ART use in our models given the limited number of events, and we were underpowered to fully discern sex differences in CVD risk (although our analysis included 35 % women). We only captured hospitalizations at the INI hospital, which could underestimate CVD morbidity in this cohort. However, as one of the largest public health reference centers for HIV care and research in Rio de Janeiro, it is also the hospital that HIV-infected persons experiencing serious hospitalizations are referred to, minimizing missing data due to hospitalizations outside of INI. It is important to note that the hospital is located within the same campus as ambulatory care and that patients’ records are linked by a unique ID. While only 10 % of the hospitalizations were randomly reviewed for adjudication, given the high observed agreement, no further quality control comparisons were conducted. Similarly, given that the Rio de Janeiro State mortality database routinely tracks vital statistics on all residents, the loss to follow up in this cohort is expected to be minimal. Lastly, evaluating the contribution of specific ART agents and classes to CVD risk is complicated by the fact that regimens typically include three or more agents, regimen changes are common, and ART use is inherently linked to virologic suppression. Important strengths of this study include the well-characterized nature of this cohort, its diversity of race and sex, use of both hospitalization and death data, and the characterization of acute CVD events beyond myocardial infarctions.

## Conclusions

Our data are the first to evaluate the association between HIV infection and ART exposure with CVD risk in Latin America. CVD in treated HIV infection is an increasingly prevalent comorbidity whose origins are not fully understood; however, traditional CVD and HIV-specific risk factors must both be considered as prevention and treatment strategies are developed. We found that traditional, virological and immunological risk factors all strongly predicted incident CVD, with ART use and subsequent virologic suppression generally associated with a decreased incidence of CVD events. Our findings support other literature suggesting an overall benefit of ART (and subsequent virologic suppression) on CVD risk [66–69]; however, our data are unique in that they document CVD events occurring in a young population with a high traditional CVD risk burden, supporting resource allocation towards risk factor reduction efforts at all ages to prevent CVD events in HIV-infected persons. Our data also highlight the importance of early HIV diagnosis and treatment, as well as the need for availability of metabolically-neutral first- and second-line ART in resource-limited settings.

## Additional files

Additional file 1: A table that details the complete cohort ART exposure history (DOCX 218 kb) Additional file 2: A table that details the process of arriving at the final multivariate regression model (DOCX 280 kb) Additional file 3: A table that details the crude incidence rate ratios for demographic/clinical characteristics and cardiovascular risk factors (DOCX 89 kb) Additional file 4: Tables that demonstrate the crude and adjusted incidence rate ratios for cardiovascular hospitalizations and deaths by exposure to specific ART agents (DOCX 128 kb) Additional file 5: Sensitivity analysis excluding VTEs, including a table that details the crude incidence rate ratios for demographic/clinical characteristics and cardiovascular risk factors (excluding VTEs), a table that details the final multivariate regression model with the exclusion of VTEs, and a table demonstrating the crude and adjusted incidence rate ratios for cardiovascular hospitalizations and deaths by exposure to specific ART agents (DOCX 199 kb)
