# Supplementary material for: Traditional and HIV-specific risk factors for cardiovascular morbidity and mortality among HIV-infected adults in Brazil: a retrospective cohort study
Source: BMC Infect Dis. 2016 Aug 8;16:376. doi: 10.1186/s12879-016-1735-4 (PMC4977901; doi:10.1186/s12879-016-1735-4)
Supplement: Additional file 1: — A table that details the complete cohort ART exposure history (DOCX 218 kb) [file 12879_2016_1735_MOESM1_ESM.docx]

**Additional Table 1: Treatment History of IPEC Cohort Exposed to Each Antiretroviral Drug and Cumulative Exposure by Cardiovascular Events 2000-2010**

|  | | **Total Study Participants** | | | **Composite CV-events** | | **CV-related hospitalizations** | | **CV-related deaths** | |
| --- | --- | --- | --- | --- | --- | --- | --- | --- | --- | --- |
|  |  | **Exposed- *n*(%)** | **PY cumulative exposure** | **Recent exposure- *n*(%)** | **Exposed- *n*(%)** | **Median cumulative years (IQR)** | **Exposed- *n*(%)** | **Median cumulative years (IQR)** | **Exposed- *n*(%)** | **Median cumulative years (IQR)** |
| **ART** | | 2960 (100%) | 18,127.24 | 2,812 (95%) | 109 (100%) | 4.55 (1.23, 8.5) | 89 (100%) | 4.55 (1.23, 8.5) | 33 (100%) | 6.13 (2.36, 9.47) |
| **NRTI** | | 2960 (100%) | 18,116.08 | 2,812 (100%) | 109 (100%) | 4.55 (1.23, 8.5) | 89 (100%) | 4.55 (1.23, 8.5) | 33 (100%) | 6.13 (2.36, 9.47) |
|  | **Lamivudine** | 2867 (97%) | 14,573.57 | 2,557 (91%) | 105 (96%) | 3.01 (0.81, 6.14) | 85 (95.5%) | 2.93 (0.81, 5.82) | 33 (100%) | 4.12 (2.2, 7.17) |
|  | **Zidovudine** | 2365 (80%) | 10,698.78 | 1,503 (53%) | 93 (85%) | 2.93 (1.3, 5.2) | 77 (86.5%) | 2.79 (0.81, 5.2) | 28 (85%) | 3.09 (1.87, 6.32) |
|  | **Tenofovir** | 1560 (53%) | 4,645.66 | 1,435 (51%) | 39 (36%) | 0.77 (0.21, 2.44) | 33 (37%) | 0.86 (0.17, 2.16) | 9 (27%) | 0.93 (0.34, 2.47) |
|  | **Stavudine** | 973 (33%) | 3,506.47 | 177 (6%) | 56 (51%) | 2.63 (1.23, 4.77) | 46 (52%) | 2.56 (1.25, 4.74) | 18 (54.5%) | 2.87 (0.88, 5.95) |
|  | **Didanosine** | 923 (31%) | 2,652.68 | 132 (5%) | 51 (47%) | 1.67 (0.91, 3.15) | 42 (47%) | 2.13 (1.07, 3.42) | 19 (58%) | 1.15 (0.45, 2.12) |
|  | **Emtricitabine** | 242 (8%) | 484.11 | 70 (2%) | 5 (5%) | 0.89 (0.37, 2.44) | 5 (6%) | 0.89 (0.37, 2.44) | 0 (0%) | 0 (0%) |
|  | **Abacavir** | 195 (7%) | 419.997 | 56 (2%) | 6 (6%) | 1.05 (0.14, 1.51) | 4 (4%) | 1.3 (0.58, 1.76) | 5 (15%) | 1.51 (1, 2.32) |
|  | **Zalcitabine** | 161 (5%) | 177.68 | 5 (0.2%) | 10 (9%) | 0.65 (0.51, 1.44) | 8 (9%) | 0.65 (0.52, 1.26) | 2 (6%) | 1.23 (0.51, 1.95) |
| **NNRTI** | | 2250 (76%) | 7,411.10 | 1,399 (50%) | 79 (72%) | 1.34 (0.47, 2.79) | 66 (74%) | 1.24 (0.50, 2.61) | 23 (70%) | 2.35 (0.21, 3.45) |
|  | **Efavirenz** | 1982 (67%) | 5,832.86 | 1,251 (44%) | 67 (61%) | 1.14 (0.31, 2.64) | 55 (62%) | 1.08 (0.34, 2.44) | 21 (64%) | 1.99 (0.31, 3.01) |
|  | **Nevirapine** | 490 (17%) | 1,300.26 | 81 (3%) | 31 (28%) | 1.07 (0.79, 1.92) | 26 (29%) | 1.06 (0.79, 1.92) | 9 (27%) | 0.88 (0.29, 1.79) |
|  | **Etravirine** | 69 (2%) | 197.07 | 56 (2%) | 1 (1%) | 1.37 | 0 (0%) | 0 | 1 (1%) | 1.37 |
|  | **Delavirdine** | 12 (0.4%) | 11.78 | 1 (0.04%) | 0 (0%) | 0 | 0 (0%) | 0 | 0 (0%) | 0 |
| **PI** | | 1824 (62%) | 9,973.03 | 1,567 (56%) | 79 (72%) | 3.54 (2.01, 6.21) | 64 (72%) | 3.39 (2.01, 5.97) | 26 (79%) | 3.98 (2.2, 6.37) |
| **Ritonavir (high dose)*** | | 581 (20%) | 1,283.55 | 86 (3%) | 35 (32%) | 0.82 (0.22, 2.2) | 25 (28%) | 0.56 (0.22, 1.11) | 14 (42%) | 1.55 (0.17, 2.84) |
| **Lopinavir/Ritonavir** | | 1052 (36%) | 2,981.27 | 658 (23%) | 41 (38%) | 1.18 (0.38, 2.69) | 36 (40%) | 1.13 (0.35, 2.77) | 12 (36%) | 2.4 (1.29, 2.81) |
| **Atazanavir** | | 809 (27%) | 2,277.88 | 571 (20%) | 22 (20%) | 1.32 (0.77, 2.73) | 21 (24%) | 1.34 (0.77, 2.73) | 2 (6%) | 0.6 (0.18, 1.02) |
|  | **Atazanavir + Ritonavir** | 627 (78%) | 1,682.66 | 507 (17%) | 13 (59%) | 0.77 (0.6, 2.41) | 13 (15%) | 0.77 (0.6, 2.41) | 0 (0%) | 0 |
|  | **Atazanavir without booster** | 311 (38%) | 595.22 | 73 (2.5%) | 13 (59%) | 1.3 (0.71, 2.08) | 12 (13%) | 1.32 (0.62, 2.22) | 2 (6%) | 0.6 (0.18, 1.02) |
| **Nelfinavir** | | 546 (18%) | 1,396.84 | 52 (2%) | 28 (27%) | 1.93 (0.99, 3.62) | 26 (29%) | 1.85 (0.97, 3.55) | 7 (21%) | 2.36 (0.75, 5.95) |
|  | **Nelfinavir without booster** | 546 (100%) | 1,388.67 | 52 (2%) | 28 (27%) | 1.93 (0.99, 3.62) | 26 (29%) | 1.85 (0.97, 3.55) | 7 (21%) | 2.36 (0.75, 5.95) |
| **Indinavir** | | 525 (18%) | 1,335.56 | 29 (1%) | 42 (39%) | 1.51 (0.84, 3.45) | 33 (37%) | 1.31 (0.73, 2.22) | 14 (42%) | 3.01 (1.42, 4.91) |
|  | **Indinavir + Ritonavir** | 33 (6%) | 85.32 | 0 (0%) | 4 (4%) | 3.05 (0.89, 4.67) | 4 (4%) | 3.05 (0.89, 4.67) | 0 (0%) | 0 |
|  | **Indinavir without booster** | 508 (97%) | 1,250.24 | 30 (1%) | 39 (36%) | 1.33 (0.80, 3.41) | 30 (34%) | 1.18 (0.58, 2.12) | 14 (42%) | 3.01 (1.42, 4.91) |
| **Saquinavir** | | 442 (15%) | 857.76 | 62 (2%) | 25 (23%) | 0.64 (0.34, 1.41) | 20 (22%) | 0.64 (0.34, 1.41) | 7 (21%) | 0.56 (0.34, 1.99) |
|  | **Saquinavir + Ritonavir** | 70 (16%) | 150.49 | 21 (0.75%) | 4 (4%) | 0.27 (0.13, 0.42) | 4 (4%) | 0.27 (0.13, 0.42) | 0 (0%) | 0 |
|  | **Saquinavir without booster** | 391 (88%) | 707.26 | 42 (1.5%) | 24 (22%) | 0.64 (0.40, 1.41) | 19 (21%) | 0.65 (0.46, 1.41) | 7 (21%) | 0.56 (0.34, 1.99) |
| **Darunavir** | | 228 (8%) | 728.53 | 218 (7%) | 6 (6%) | 1.3 (0.68, 1.49) | 4 (4%) | 1.36 (0.63, 2.71) | 3 (9%) | 1.37 (0.68, 1.84) |
|  | **Darunavir + Ritonavir** | 220 (96%) | 692.89 | 212 (7.5%) | 6 (6%) | 1.3 (0.68, 1.49) | 4 (4%) | 1.36 (0.63, 2.71) | 3 (9%) | 1.37 (0.68, 1.84) |
| **Amprenavir** | | 102 (3%) | 160 | 7 (0.25%) | 6 (6%) | 1.25 (0.79, 1.52) | 5 (6%) | 1.13 (0.79, 1.37) | 1 (1%) | 1.97 |
|  | **Amprenavir + Ritonavir** | 32 (31%) | 45.84 | 0 (0%) | 3 (3%) | 0.84 (0.79, 1.97) | 2 (2%) | 0.81 (0.79, 0.84) | 1 (1%) | 1.97 |
|  | **Amprenavir without booster** | 75 (74%) | 114.15 | 7 (0.25%) | 4 (4%) | 0.83 (0.15, 1.44) | 4 (4%) | 0.83 (0.15, 1.44) | 0 (0%) | 0 |
| **Fosamprenavir** | | 70 (2%) | 83.02 | 56 (2%) | 0 (0%) | 0 | 0 (0%) | 0 | 0 (0%) | 0 |
|  | **Fosamprenavir + Ritonavir** | 68 (97%) | 79.53 | 55 (2%) | 0 (0%) | 0 | 0 (0%) | 0 | 0 (0%) | 0 |
| **Tipranavir** | | 39 (1%) | 74.19 | 10 (0.4%) | 2 (2%) | 2.054 (0.92, 3.19) | 2 (2%) | 2.05 (0.92, 3.19) | 1 (3%) | 4.59 |
|  | **Tipranavir + Ritonavir** | 20 (51%) | 54.01 | 7 (0.25%) | 2 (2%) | 2.05 (0.92, 3.19) | 2 (2%) | 2.05 (0.92, 3.19) | 1 (1%) | 4.59 |
|  | **Tipranavir without booster** | 19 (49%) | 20.18 | 3 (0.11%) | 0 (0%) | 0 | 0 (0%) | 0 | 0 (0%) | 0 |
| **Integrase Inhibitor** | | 180 (6%) | 363.75 | 172 (6%) | 4 (4%) | 1.29 (0.47, 3.43) | 4 (4%) | 1.29 (0.47, 3.43) | 0 (0%) | 0 |
| **Fusion Inhibitor** | | 111 (4%) | 236.14 | 41 (1.5%) | 4 (4%) | 0.92 (0.36, 1.95) | 3 (3%) | 1.15 (0.04, 2.75) | 2 (6%) | 0.34 (0, 0.68) |
| **Entry Inhibitor** | | 42 (1%) | 78.08 | 34 (1%) | 0 (0%) | 0 | 0 (0%) | 0 | 0 (0%) | 0 |
