# Supplementary material for: Traditional and HIV-specific risk factors for cardiovascular morbidity and mortality among HIV-infected adults in Brazil: a retrospective cohort study
Source: BMC Infect Dis. 2016 Aug 8;16:376. doi: 10.1186/s12879-016-1735-4 (PMC4977901; doi:10.1186/s12879-016-1735-4)
Supplement: Additional file 2: — A table that details the process of arriving at the final multivariate regression model (DOCX 280 kb) [file 12879_2016_1735_MOESM2_ESM.docx]

**Additional Table 2. Process of arriving at the final multivariate regression model for the primary and secondary outcomes**

| **Multivariate Regression Model for Composite Outcome** | | | | | | | | | | | | | | | | | | | | | | | | | | | |
| --- | --- | --- | --- | --- | --- | --- | --- | --- | --- | --- | --- | --- | --- | --- | --- | --- | --- | --- | --- | --- | --- | --- | --- | --- | --- | --- | --- |
|  | **Model 1- All Covariates** | | | **Model 2-Dropped IDU, heavy cocaine** | | | | | | **Model 3- Additionally dropped weight** | | | | | | **Model 4- Additionally dropped education** | | | | | | **Model 5 (FINAL MODEL)- Drop if p>0.1** | | | | | |
| **Covariates** | **IRR** | **P-value** | **95% CI** | **IRR** | | **P-value** | | **95% CI** | | **IRR** | | **P-value** | | **95% CI** | | **IRR** | | **P-value** | | **95% CI** | | **IRR** | | **P-value** | | **95% CI** | |
| Age ≥ 40 years | 2.16 | <0.001 | 1.43-3.27 | 2.21 | | <0.001 | | 1.46-3.33 | | 2.23 | | <0.001 | | 1.48-3.36 | | 2.17 | | <0.001 | | 1.44-3.27 | | 2.3 | | <0.001 | | 1.54-3.45 | |
| Male gender | 1.80 | 0.008 | 1.17-2.76 | 1.76 | | 0.01 | | 1.14-2.69 | | 1.53 | | 0.046 | | 1.01-2.33 | | 1.45 | | 0.08 | | 0.96-2.20 | | 1.49 | | 0.061 | | 0.98-2.25 | |
| Non-white race | 1.45 | 0.069 | 0.97-2.16 | 1.43 | | 0.08 | | 0.96-2.14 | | 1.43 | | 0.079 | | 0.96-2.14 | | 1.59 | | 0.02 | | 1.08-2.34 | | 1.56 | | 0.024 | | 1.06-2.30 | |
| ≤8 yrs education | 1.46 | 0.079 | 0.96-2.22 | 1.42 | | 0.10 | | 0.94-2.16 | | 1.49 | | 0.061 | | 0.98-2.26 | | - | | - | | - | | - | | - | | - | |
| IDU | 1.43 | 0.56 | 0.43-4.71 | - | | - | | - | | - | | - | | - | | - | | - | | - | | - | | - | | - | |
| Heavy cocaine use | 0.65 | 0.30 | 0.29-1.47 | - | | - | | - | | - | | - | | - | | - | | - | | - | | - | | - | | - | |
| Nadir CD4+ cell count ≤ 50 | 1.66 | 0.022 | 1.08-2.55 | 1.65 | | 0.022 | | 1.07-2.54 | | 1.73 | | 0.011 | | 1.13-2.65 | | 1.74 | | 0.011 | | 1.13-2.66 | | 1.73 | | 0.011 | | 1.13-2.64 | |
| Detectable HIV-1 RNA | 3.24 | <0.001 | 2.03-5.16 | 3.21 | | <0.001 | | 2.01-5.12 | | 3.25 | | <0.001 | | 2.06-5.12 | | 3.30 | | <0.001 | | 2.09-5.19 | | 3.39 | | <0.001 | | 2.16-5.32 | |
| % time with HIV on tx | 0.40 | 0.009 | 0.20-0.79 | 0.40 | | 0.009 | | 0.20-0.80 | | 0.37 | | 0.005 | | 0.19-0.75 | | 0.37 | | 0.005 | | 0.18-0.74 | | 0.36 | | 0.004 | | 0.18-0.72 | |
| Hx diabetes mellitus | 1.57 | 0.091 | 0.93-2.63 | 1.56 | | 0.092 | | 0.93-2.62 | | 1.47 | | 0.15 | | 0.87-2.46 | | 1.42 | | 0.19 | | 0.85-2.38 | | - | | - | | - | |
| Hx of hypertension | 1.65 | 0.018 | 1.09-2.50 | 1.64 | | 0.02 | | 1.08-2.48 | | 1.49 | | 0.059 | | 0.98-2.25 | | 1.5 | | 0.054 | | 0.99-2.27 | | 1.56 | | 0.033 | | 1.04-2.34 | |
| Hx of dyslipidemia | 0.73 | 0.13 | 0.48-1.10 | 0.73 | | 0.13 | | 0.48-1.10 | | 0.69 | | 0.081 | | 0.46-1.05 | | 0.69 | | 0.078 | | 0.46-1.04 | | 0.70 | | 0.087 | | 0.47-1.05 | |
| Prior CVD | 2.41 | 0.004 | 1.33-4.36 | 2.42 | | 0.004 | | 1.33-4.37 | | 2.74 | | 0.001 | | 1.52-4.93 | | 2.67 | | 0.001 | | 1.48-4.82 | | 2.82 | | <0.001 | | 1.58-5.05 | |
| Ever smoked | 1.36 | 0.21 | 0.84-2.20 | 1.36 | | 0.21 | | 0.84-2.20 | | 1.41 | | 0.16 | | 0.87-2.28 | | 1.44 | | 0.14 | | 0.89-2.31 | | - | | - | | - | |
| Weight tertile, 1st * | 1.43 | 0.13 | 0.90-2.28 | 1.44 | | 0.121 | | 0.91-2.29 | | - | | - | | - | | - | | - | | - | | - | | - | | - | |
| Weight tertile, 3rd * | 0.65 | 0.095 | 0.39-1.08 | 0.64 | | 0.092 | | 0.29-1.07 | | - | | - | | - | | - | | - | | - | | - | | - | | - | |
| Calendar year cohort entry | 1.00 | 0.21 | 0.84-2.21 | 1.00 | | 0.91 | | 0.96-1.03 | | 1.00 | | 0.87 | | 0.97-1.04 | | 1.00 | | 0.89 | | 0.97-1.04 | | - | | - | | - | |
| **Multivariate Regression Model for Hospitalizations** | | | | | | | | | | | | | | | | | | | | | | | | | | | |
|  | **Model 1- All Covariates** | | | **Model 2-Dropped IDU, heavy cocaine** | | | | | | **Model 3- Additionally dropped weight** | | | | | | **Model 4- Additionally dropped education** | | | | | | **Model 5 (FINAL MODEL)- Drop if p>0.1** | | | | | |
| **Covariates** | **IRR** | **P-value** | **95% CI** | **IRR** | | **P-value** | | **95% CI** | | **IRR** | | **P-value** | | **95% CI** | | **IRR** | | **P-value** | | **95% CI** | | **IRR** | | **P-value** | | **95% CI** | |
| Age ≥ 40 years | 2.07 | 0.002 | 1.32-3.24 | 2.11 | | 0.001 | | 1.35-3.30 | | 2.13 | | 0.001 | | 1.36-3.33 | | 2.07 | | 0.001 | | 1.32-3.23 | | 2.17 | | 0.001 | | 1.40-3.37 | |
| Male gender | 2.27 | 0.001 | 1.38-3.71 | 2.22 | | 0.001 | | 1.36-3.61 | | 1.89 | | 0.009 | | 1.17-3.05 | | 1.79 | | 0.016 | | 1.11-2.87 | | 1.80 | | 0.015 | | 1.12-2.88 | |
| Non-white race | 1.59 | 0.041 | 1.02-2.47 | 1.56 | | 0.050 | | 1.00-2.42 | | 1.54 | | 0.055 | | 0.99-2.40 | | 1.72 | | 0.014 | | 1.12-2.64 | | 1.68 | | 0.017 | | 1.10-2.58 | |
| IDU | 1.71 | 0.38 | 0.52-5.70 | - | | - | | - | | - | | - | | - | | - | | - | | - | | - | | - | | - | |
| Heavy cocaine use | 0.63 | 0.31 | 0.26-1.53 | - | | - | | - | | - | | - | | - | | - | | - | | - | | - | | - | | - | |
| Nadir CD4+ cell count ≤ 50 | 1.72 | 0.023 | 1.08-2.75 | 1.71 | | 0.024 | | 1.07-2.73 | | 1.79 | | 0.014 | | 1.13-2.84 | | 1.79 | | 0.014 | | 1.13-2.85 | | 1.77 | | 0.016 | | 1.11-2.81 | |
| Viral suppression | 3.35 | <0.001 | 1.97-5.70 | 3.31 | | <0.001 | | 1.94-5.63 | | 3.35 | | <0.001 | | 1.99-5.64 | | 3.41 | | <0.001 | | 2.03-5.72 | | 3.48 | | <0.001 | | 2.08-5.84 | |
| % time with HIV on tx | 0.39 | 0.015 | 0.18-0.83 | 0.39 | | 0.016 | | 0.18-0.84 | | 0.36 | | 0.009 | | 0.17-0.78 | | 0.35 | | 0.008 | | 0.16-0.77 | | 0.37 | | 0.011 | | 0.17-0.80 | |
| Hx diabetes mellitus | 1.87 | 0.029 | 1.06-3.28 | 1.86 | | 0.031 | | 1.06-3.27 | | 1.73 | | 0.056 | | 0.99-3.02 | | 1.68 | | 0.069 | | 0.96-2.95 | | 1.71 | | 0.018 | | 1.10-2.66 | |
| Hx of hypertension | 1.79 | 0.012 | 1.13-2.81 | 1.78 | | 0.013 | | 1.13-2.80 | | 1.59 | | 0.044 | | 1.01-2.49 | | 1.60 | | 0.042 | | 1.02-2.51 | | 0.64 | | 0.043 | | 0.41-0.99 | |
| Hx of dyslipidemia | 0.61 | 0.036 | 0.39-0.97 | 0.62 | | 0.038 | | 0.39-0.97 | | 0.59 | | 0.022 | | 0.37-0.92 | | 0.59 | | 0.021 | | 0.37-0.92 | | 1.86 | | 0.099 | | 0.89-3.87 | |
| Prior CVD | 1.51 | 0.28 | 0.71-3.21 | 1.52 | | 0.28 | | 0.71-3.21 | | 1.79 | | 0.13 | | 0.85-3.75 | | 1.73 | | 0.15 | | 0.82-3.63 | | - | | - | | - | |
| Ever smoked | 1.02 | 0.95 | 0.62-1.67 | 1.02 | | 0.95 | | 0.62-1.67 | | 1.07 | | 0.80 | | 0.65-1.75 | | 1.09 | | 0.74 | | 0.66-1.78 | | - | | - | | - | |
| Weight tertile, 1st * | 1.45 | 0.16 | 0.87-2.42 | 1.46 | | 0.15 | | 0.87-2.43 | | - | | - | | - | | - | | - | | - | | - | | - | | - | |
| Weight tertile, 3rd * | 0.60 | 0.080 | 0.34-1.06 | 0.6 | | 0.077 | | 0.34-1.06 | | - | | - | | - | | - | | - | | - | | - | | - | | - | |
| Calendar year cohort entry | 0.99 | 0.57 | 0.95-1.03 | 0.98 | | 0.43 | | 0.95-1.02 | | 0.99 | | 0.64 | | 0.95-1.03 | | 0.99 | | 0.60 | | 0.95-1.03 | | - | | - | | - | |
| **Multivariate Regression Model for Deaths** | | | | | | | | | | | | | | | | | | | | | | | | | | | |
|  | **Model 1- All Covariates** | | | | **Model 2-Combined CV risk factors** | | | | | | **Model 3- Drop IDU, heavy cocaine** | | | | | | **Model 4- Additionally dropped weight** | | | | | | **Model 5 (FINAL MODEL)- Drop if p>0.1** | | | | |
| **Covariates** | **IRR** | **P-value** | **95% CI** | | **IRR** | | **P-value** | | **95% CI** | | **IRR** | | **P-value** | | **95% CI** | | **IRR** | | **P-value** | | **95% CI** | | **IRR** | | **P-value** | | **95% CI** |
| Age ≥ 40 years | 2.45 | 0.025 | 1.12-5.34 | | 3.00 | | 0.004 | | 1.43-6.29 | | 3.14 | | 0.002 | | 1.50-6.56 | | 3.14 | | 0.002 | | 1.50-6.56 | | 3.17 | | 0.002 | | 1.52-6.59 |
| Male gender | 1.24 | 0.58 | 0.58-2.65 | | 1.50 | | 0.29 | | 0.70-3.21 | | 1.46 | | 0.32 | | 0.69-3.10 | | 1.25 | | 0.55 | | 0.61-2.58 | | - | | - | | - |
| Non-white race | 1.51 | 0.27 | 0.73-3.11 | | 1.49 | | 0.27 | | 0.73-3.04 | | 1.46 | | 0.30 | | 0.72-2.98 | | 1.46 | | 0.30 | | 0.72-2.98 | | - | | - | | - |
| ≤8 yrs education | 2.37 | 0.035 | 1.06-5.30 | | 2.16 | | 0.061 | | 0.97-4.82 | | 1.08 | | 0.074 | | 0.93-4.63 | | 2.15 | | 0.063 | | 0.96-4.79 | | 2.30 | | 0.033 | | 1.07-4.97 |
| IDU | 2.75 | 0.35 | 0.33-22.66 | | 1.88 | | 0.55 | | 0.24-15.05 | | - | | - | | - | | - | | - | | - | | - | | - | | - |
| Heavy cocaine use | 0.34 | 0.31 | 0.04-2.75 | | 0.36 | | 0.33 | | 0.04-2.86 | | - | | - | | - | | - | | - | | - | | - | | - | | - |
| Nadir CD4+ cell count ≤ 50 | 2.13 | 0.048 | 1.01-4.51 | | 1.74 | | 0.14 | | 0.84-3.59 | | 1.72 | | 0.15 | | 0.83-3.55 | | 1.75 | | 0.13 | | 0.85-3.60 | | - | | - | | - |
| Detectable HIV-1 RNA | 2.43 | 0.024 | 1.12-5.24 | | 2.47 | | 0.019 | | 1.16-5.28 | | 2.44 | | 0.021 | | 1.14-5.21 | | 2.56 | | 0.015 | | 1.20-5.45 | | 2.85 | | 0.005 | | 1.37-5.94 |
| % time with HIV on tx | 0.99 | 0.97 | 0.56-1.75 | | 1.00 | | 0.996 | | 0.61-1.65 | | 1.01 | | 0.97 | | 0.63-1.62 | | 1.00 | | 0.99 | | 0.61-1.63 | | - | | - | | - |
| Hx diabetes mellitus | 0.68 | 0.45 | 0.24-1.88 | | - | | - | | - | | - | | - | | - | | - | | - | | - | | - | | - | | - |
| Hx of hypertension | 1.66 | 0.21 | 0.76-3.63 | | - | | - | | - | | - | | - | | - | | - | | - | | - | | - | | - | | - |
| Hx of dyslipidemia | 0.89 | 0.77 | 0.41-1.93 | | - | | - | | - | | - | | - | | - | | - | | - | | - | | - | | - | | - |
| Prior CVD | 7.38 | <0.001 | 3.14-17.39 | | - | | - | | - | | - | | - | | - | | - | | - | | - | | - | | - | | - |
| Combined CV risk factors | - | - | - | | 3.91 | | 0.012 | | 1.35-11.34 | | 3.91 | | 0.012 | | 1.35-11.36 | | 3.75 | | 0.015 | | 1.30-10.87 | | 3.85 | | 0.012 | | 1.35-11.04 |
| Weight tertile, 1st * | 2.01 | 0.13 | 0.82-4.93 | | 2.18 | | 0.085 | | 0.90-5.29 | | 2.19 | | 0.083 | | 0.90-5.31 | | - | | - | | - | | - | | - | | - |
| Weight tertile, 3rd * | 1.27 | 0.13 | 0.82-4.93 | | 1.16 | | 0.75 | | 0.45-2.99 | | 1.13 | | 0.79 | | 0.44-2.911 | | - | | - | | - | | - | | - | | - |
| Calendar year cohort entry | 1.00 | 0.95 | 0.94-1.07 | | 0.99 | | 0.95 | | 0.93-1.06 | | 1.00 | | 0.95 | | 0.94-1.07 | | 0.99 | | 0.81 | | 0.93-1.06 | | - | | - | | - |
