# Supplementary material for: Traditional and HIV-specific risk factors for cardiovascular morbidity and mortality among HIV-infected adults in Brazil: a retrospective cohort study
Source: BMC Infect Dis. 2016 Aug 8;16:376. doi: 10.1186/s12879-016-1735-4 (PMC4977901; doi:10.1186/s12879-016-1735-4)
Supplement: Additional file 3: — A table that details the crude incidence rate ratios for demographic/clinical characteristics and cardiovascular risk factors (DOCX 89 kb) [file 12879_2016_1735_MOESM3_ESM.docx]

**Supplementary Table 3. Crude Incidence Rate Ratios for Demographic/Clinical Characteristics and Cardiovascular Risk Factors.**

|  | | **Composite CVD related end points** | | | | | **CVD-related Hospitalizations** | | | | | | **CVD-related death** | | | | |
| --- | --- | --- | --- | --- | --- | --- | --- | --- | --- | --- | --- | --- | --- | --- | --- | --- | --- |
|  |  | **cIRR** | **95% CI** | | **p-value** | | **cIRR** | | **95% CI** | | **p-value** | | **cIRR** | | **95% CI** | | **p-value** |
| **Participants characteristics** | |  |  | |  | |  | |  | |  | |  | | | | |
| Age ≥ 40 years | | **2.37** | **1.62-3.47** | | **<0.001** | | **2.14** | | **1.41-3.25** | | **<0.001** | | **3.31** | | **1.60-6.82** | | **0.001** |
| Male gender | | 1.35 | 0.90-2.03 | | 0.15 | | 1.57 | | 0.99-2.49 | | 0.056 | | 1.06 | | 0.52-2.16 | | 0.86 |
| Non-white race | | **1.68** | **1.16-2.45** | | **0.007** | | **1.81** | | **1.20-2.76** | | **0.005** | | 1.75 | | 0.88-3.50 | | 0.11 |
| ≤8 yrs education | | **1.64** | **1.11-2.42** | | **0.013** | | **1.69** | | **1.10-2.61** | | **0.018** | | **2.51** | | **1.17-5.41** | | **0.018** |
| IDU exposure | | 1.10 | 0.35-3.47 | | 0.87 | | 1.36 | | 0.43-4.29 | | 0.60 | | 1.22 | | 0.17-8.90 | | 0.85 |
| Cocaine use | | 0.87 | 0.40-1.87 | | 0.72 | | 0.92 | | 0.40-2.10 | | 0.83 | | 0.39 | | 0.05-2.88 | | 0.36 |
| Nadir CD4+ T-cell count ≤50cells/mm3* | | **2.04** | **1.36-3.06** | | **0.001** | | **2.17** | | **1.39-3.37** | | **0.001** | | **2.31** | | **1.14-4.67** | | **0.020** |
| Detectable HIV-1 RNA* | | **3.99** | **2.61-6.11** | | **<0.001** | | **4.14** | | **2.56-6.70** | | **<0.001** | | **2.91** | | **1.37-6.20** | | **0.005** |
| Viremia copy-years, log10copyXy/mL | | 1.02 | 0.81-1.29 | | 0.84 | | 1.11 | | 0.85-1.43 | | 0.45 | | 0.77 | | 0.52-1.14 | | 0.20 |
| Time on ART/Years with known HIV | | **0.29** | **0.15-0.55** | | **<0.001** | | **0.27** | | **0.14-0.58** | | **0.001** | | 1.03 | | 0.69-1.55 | | 0.87 |
| **Cardiovascular risk factors** | | | | | | | | | | | | | | | | | |
| History of diabetes mellitus | | **1.68** | **1.04-2.71** | **0.033** | | **1.79** | | **1.06-3.00** | | **0.028** | | 1.21 | | 0.47-3.13 | | 0.70 | |
| History of hypertension | | **1.87** | **1.29-2.73** | **0.001** | | **1.83** | | **1.21-2.78** | | **0.004** | | **2.68** | | **1.34-5.34** | | **0.005** | |
| History of dyslipidemia | | 0.77 | 0.53-1.12 | 0.18 | | 0.66 | | 0.44-1.00 | | 0.052 | | 1.17 | | 0.58-2.38 | | 0.66 | |
| History of chronic kidney disease | | 1.59 | 0.39-6.45 | 0.52 | | 1.96 | | 0.48-7.95 | | 0.35 | | 2.27 | | 0.31-16.6 | | 0.42 | |
| >1 metabolic risk factors for CVD | | 1.09 | 0.71-1.66 | 0.69 | | 0.95 | | 0.60-1.49 | | 0.82 | | **2.92** | | **1.03-8.32** | | **0.044** | |
| Prior CVD | | **3.99** | **2.35-6.79** | **<0.001** | | **2.61** | | **1.31-5.2** | | **0.006** | | **9.99** | | **4.85-20.61** | | **<0.001** | |
| Ever smoked | | **2.04** | **1.28-3.26** | **0.003** | | 1.59 | | 0.97-2.59 | | 0.065 | | - | | - | | - | |
| Weight tertiles*  *Missing nadir CD4, viral suppression and weight values filled in using multiple imputation | Lowest third | **1.67** | **1.07-2.62** | **0.024** | | 1.56 | | 0.96-2.54 | | 0.074 | | 2.12 | | 0.89-5.06 | | 0.091 | |
|  | Highest third | 0.76 | 0.46-1.27 | 0.29 | | 0.72 | | 0.41-1.24 | | 0.24 | | 1.17 | | 0.46-2.97 | | 0.74 | |
