# Supplementary material for: Traditional and HIV-specific risk factors for cardiovascular morbidity and mortality among HIV-infected adults in Brazil: a retrospective cohort study
Source: BMC Infect Dis. 2016 Aug 8;16:376. doi: 10.1186/s12879-016-1735-4 (PMC4977901; doi:10.1186/s12879-016-1735-4)
Supplement: Additional file 4: — Tables that demonstrate the crude and adjusted incidence rate ratios for cardiovascular hospitalizations and deaths by exposure to specific ART agents (DOCX 128 kb) [file 12879_2016_1735_MOESM4_ESM.docx]

| **Supplementary Table 4: Crude and Adjusted* Incidence Rate Ratios for Cardiovascular Hospitalizations by Exposure to Specific Antiretroviral Drugs** | | | | | | | | | | | | | |  |
| --- | --- | --- | --- | --- | --- | --- | --- | --- | --- | --- | --- | --- | --- | --- |
|  |  | **Cumulative Exposure** | | | | | | **Recent Exposure** | | | | | | |
|  |  | **cIRR** | **95% CI** | **P** | **aIRR** | **95% CI** | **P** | **cIRR** | **95% CI** | **P** | **aIRR** | **95% CI** | **P** | |
| **ART** | | **0.84** | **0.80-0.89** | **<0.001** | **0.78** | **0.74-0.83** | **<0.001** | - | - | - | - | - | - | |
| **NRTIs** | | **0.84** | **0.80-0.89** | **<0.001** | **0.78** | **0.73-0.83** | **<0.001** | - | - | - | - | - | - | |
| Lamivudine | | **0.78** | **0.73-0.83** | **<0.001** | **0.75** | **0.69-0.80** | **<0.001** | **0.50** | **0.26-0.96** | **0.038** | 0.66 | 0.33-1.30 | 0.23 | |
| Zidovudine | | **0.86** | **0.80-0.91** | **<0.001** | **0.83** | **0.77-0.89** | **<0.001** | 1.32 | 0.85-2.05 | 0.21 | 1.44 | 0.93-2.24 | 0.10 | |
| Tenofovir | | **0.65** | **0.55-0.77** | **<0.001** | **0.61** | **0.51-0.73** | **<0.001** | **0.42** | **0.27-0.67** | **<0.001** | **0.35** | **0.22-0.56** | **<0.001** | |
| Stavudine | | 0.97 | 0.89-1.06 | 0.48 | 0.95 | 0.86-1.04 | 0.26 | **6.33** | **3.87-10.36** | **<0.001** | **5.15** | **2.98-8.87** | **<0.001** | |
| Didanosine | | 0.97 | 0.88-1.07 | 0.60 | 0.93 | 0.83-1.04 | 0.18 | 1.55 | 0.67-3.55 | 0.303 | 1.40 | 0.60-3.29 | 0.43 | |
| Emtricitabine | | 0.84 | 0.53-1.33 | 0.46 | 0.91 | 0.56-1.47 | 0.70 | 2.82 | 0.89-8.92 | 0.078 | 3.26 | 1.00-10.60 | 0.05 | |
| Abacavir | | 0.70 | 0.41-1.20 | 0.19 | 0.61 | 0.32-1.15 | 0.13 | 1.19 | 0.29-4.84 | 0.81 | 0.78 | 0.19-3.20 | 0.73 | |
| Zalcitabine | | 0.96 | 0.57-1.61 | 0.88 | 0.89 | 0.52-1.55 | 0.69 | - | - | - | - | - | - | |
| **NNRTIs** | | **0.75** | **0.68-0.84** | **<0.001** | **0.76** | **0.68-0.85** | **<0.001** | 1.03 | 0.67-1.57 | 0.90 | 1.40 | 0.90-2.21 | 0.14 | |
| Efavirenz | | **0.75** | **0.66-0.85** | **<0.001** | **0.77** | **0.67-0.88** | **<0.001** | 0.98 | 0.63-1.51 | 0.92 | 1.40 | 0.88-2.24 | 0.16 | |
| Nevirapine | | 0.92 | 0.79-1.07 | 0.28 | 0.9 | 0.76-1.07 | 0.25 | 1.84 | 0.75-4.55 | 0.19 | 2.31 | 0.91-5.82 | 0.077 | |
| **PIs** | | **0.92** | **0.87-0.97** | **0.001** | **0.87** | **0.81-0.92** | **<0.001** | 1.25 | 0.79-1.96 | 0.34 | 0.79 | 0.48-1.30 | 0.36 | |
| **PIs (without high dose RTV)** | | **0.92** | **0.87-0.97** | **0.001** | **0.86** | **0.81-0.92** | **<0.001** | 1.25 | 0.80-1.96 | 0.33 | 0.79 | 0.48-1.30 | 0.36 | |
| Ritonavir | | 0.81 | 0.65-1.01 | 0.06 | **0.71** | **0.54-0.93** | **0.014** | 2.11 | 0.77-5.75 | 0.15 | 1.29 | 0.46-3.60 | 0.62 | |
| Lopinavir/Ritonavir | | **0.87** | **0.77-0.98** | **0.03** | **0.81** | **0.70-0.93** | **0.004** | 1.38 | 0.87-2.17 | 0.17 | 1.09 | 0.68-1.74 | 0.72 | |
| Atazanavir + Ritonavir | | **0.64** | **0.49-0.85** | **0.002** | **0.65** | **0.49-0.86** | **0.003** | 0.45 | 0.23-0.87 | 0.018 | **0.48** | **0.25-0.94** | **0.032** | |
| Darunavir + Ritonavir | | 0.70 | 0.46-1.06 | 0.093 | **0.62** | **0.40-0.95** | **0.03** | 0.52 | 0.19-1.41 | 0.20 | **0.34** | **0.12-0.93** | **0.036** | |
| Nelfinavir without booster | | 0.99 | 0.88-1.11 | 0.86 | 0.96 | 0.84-1.10 | 0.59 | **8.63** | **4.33-17.21** | **<0.001** | **7.03** | **2.78-17.81** | **<0.001** | |
| Indinavir without booster | | 0.99 | 0.87-1.13 | 0.92 | 0.92 | 0.80-1.07 | 0.28 | **9.25** | **4.04-21.21** | **<0.001** | 1.12 | 0.15-8.17 | 0.91 | |
| Saquinavir + Ritonavir | | 0.61 | 0.18-2.07 | 0.43 | 0.52 | 0.12-2.17 | 0.37 | 1.99 | 0.28-14.33 | 0.49 | 1.97 | 0.60-6.54 | 0.27 | |
| Saquinavir without booster | | 0.84 | 0.63-1.13 | 0.25 | 0.73 | 0.50-1.05 | 0.091 | 3.62 | 1.14-11.44 | 0.029 | **7.03** | **2.78-17.81** | **<0.001** | |
| Amprenavir + Ritonavir | | 1.22 | 0.45-3.34 | 0.70 | 1.03 | 0.33-3.21 | 0.96 | - | - | - | - | - | - | |
| Amprenavir without booster | | 0.92 | 0.48-1.79 | 0.82 | 0.72 | 0.31-1.63 | 0.43 | **11.77** | **1.64-84.54** | **0.014** | 4.77 | 0.63-35.98 | 0.13 | |
| **Integrase Inhibitors** | | 0.89 | 0.58-1.37 | 0.60 | 0.82 | 0.53-1.27 | 0.37 | 0.77 | 0.28-2.09 | 0.60 | 0.60 | 0.22-1.66 | 0.32 | |

*Final model was adjusted for age ≥ 40 years, male gender, non-white race, nadir CD4+ T lymphocyte count ≤50cells/mm3, viral suppression, %of time with HIV on ART,

history of diabetes mellitus, history of hypertension, history of dyslipidemia, and calendar year of cohort entry

**Supplementary Table 5: Crude and Adjusted* Incidence Rate Ratios for Cardiovascular Deaths by Exposure to Specific Antiretroviral Drugs**

|  |  | **Cumulative Exposure** | | | | | | **Recent Exposure** | | | | | |
| --- | --- | --- | --- | --- | --- | --- | --- | --- | --- | --- | --- | --- | --- |
|  |  | **cIRR** | **95% CI** | **P** | **aIRR** | **95% CI** | **P** | **cIRR** | **95% CI** | **P** | **aIRR** | **95% CI** | **P** |
| **ART** | | **0.88** | **0.82-0.96** | **0.002** | **0.78** | **0.71-0.86** | **<0.001** | - | - | - | - | - | - |
| **NRTIs** | | **0.88** | **0.82-0.96** | **0.002** | **0.78** | **0.71-0.86** | **<0.001** | - | - | - | - | - | - |
| Lamivudine | | **0.86** | **0.79-0.95** | **0.002** | **0.80** | **0.72-0.89** | **<0.001** | 0.55 | 0.17-1.82 | 0.33 | 0.67 | 0.20-2.26 | 0.52 |
| Zidovudine | | **0.88** | **0.80-0.98** | **0.016** | **0.82** | **0.73-0.92** | **0.001** | 0.90 | 0.45-1.80 | 0.76 | 0.86 | 0.43-1.73 | 0.67 |
| Tenofovir | | **0.62** | **0.46-0.84** | **0.002** | **0.60** | **0.44-0.82** | **0.001** | **0.31** | **0.14-0.69** | **0.004** | **0.29** | **0.13-0.66** | **0.003** |
| Stavudine | | 1.01 | 0.89-1.14 | 0.89 | 0.97 | 0.84-1.12 | 0.70 | **6.54** | **2.94-14.56** | **<0.001** | **5.47** | **2.39-12.49** | **<0.001** |
| Didanosine | | 0.88 | 0.72-1.08 | 0.23 | 0.79 | 0.62-1.00 | 0.052 | 2.15 | 0.65-7.04 | 0.22 | 1.50 | 0.66-3.45 | 0.34 |
| Abacavir | | 1.00 | 0.74-1.36 | 0.99 | 1.01 | 0.73-1.40 | 0.96 | 3.33 | 0.80-13.93 | 0.10 | 1.89 | 0.44-8.13 | 0.39 |
| Zalcitabine | | 0.97 | 0.42-2.24 | 0.94 | 0.99 | 0.42-2.30 | 0.97 | - | - | - | - | - | - |
| **NNRTIs** | | **0.79** | **0.68-0.93** | **0.004** | **0.80** | **0.68-0.94** | **0.006** | 1.01 | 0.50-2.04 | 0.97 | 1.54 | 0.74-3.21 | 0.25 |
| Efavirenz | | **0.82** | **0.69-0.97** | **0.018** | **0.84** | **0.71-0.99** | **0.039** | 0.75 | 0.35-1.58 | 0.45 | 1.07 | 0.49-2.35 | 0.86 |
| Nevirapine | | 0.87 | 0.65-1.16 | 0.35 | 0.83 | 0.60-1.13 | 0.24 | 3.22 | 0.98-10.56 | 0.054 | **4.74** | **1.41-15.94** | **0.012** |
| **PIs** | | 0.95 | 0.88-1.03 | 0.21 | **0.90** | **0.82-0.98** | **0.018** | 1.14 | 0.55-2.37 | 0.72 | 0.69 | 0.32-1.52 | 0.36 |
| **PIs (without high dose RTV)** | | 0.94 | 0.87-1.02 | 0.17 | **0.89** | **0.81-0.98** | **0.014** | 1.00 | 0.49-2.05 | 1.00 | 0.60 | 0.28-1.29 | 0.19 |
| Ritonavir | | 1.04 | 0.87-1.26 | 0.65 | 1.00 | 0.82-1.22 | 0.99 | **8.49** | **3.27-22.04** | **<0.001** | **5.76** | **2.16-15.39** | **<0.001** |
| Atazanavir | | 0.23 | 0.04-1.17 | 0.076 | 0.17 | 0.03-1.10 | 0.063 | **0.097** | **0.013-0.71** | **0.022** | **0.092** | **0.01-0.67** | **0.019** |
| Boosted Lopinavir | | 0.89 | 0.74-1.08 | 0.25 | 0.84 | 0.69-1.03 | 0.10 | 0.99 | 0.45-2.21 | 0.99 | 0.70 | 0.31-1.59 | 0.40 |
| Boosted Darunavir | | 0.79 | 0.47-1.33 | 0.38 | 0.77 | 0.45-1.31 | 0.33 | 1.08 | 0.33-3.54 | 0.90 | 0.93 | 0.28-3.09 | 0.91 |
| Boosted Amprenavir | | 1.93 | 0.85-4.38 | 0.12 | 2.07 | 0.81-5.26 | 0.13 | - | - | - | - | - | - |
| Unboosted Nelfinavir | | 0.97 | 0.78-1.19 | 0.75 | 0.92 | 0.73-1.17 | 0.50 | **8.03** | **2.45-26.35** | **0.001** | **6.66** | **1.92-23.07** | **0.003** |
| Unboosted Indinavir | | **1.17** | **1.04-1.32** | **0.011** | 1.11 | 0.97-1.27 | 0.14 | **17.44** | **6.12-49.71** | **<0.001** | **16.79** | **5.05-55.8** | **<0.001** |
| Unboosted Saquinavir | | 0.99 | 0.72-1.37 | 0.95 | 0.94 | 0.67-1.32 | 0.73 | **6.70** | **1.60-28.05** | **0.009** | **4.57** | **1.07-19.61** | **0.041** |
| **Integrase Inhibitors** | | - | - | - | - | - | - | 0.50 | 0.07-3.66 | 0.50 | 0.46 | 0.06-3.37 | 0.44 |

*Final model was adjusted for age ≥ 40 years, ≤ 8 years of education, viral suppression, combined cardiovascular risk factors (history of diabetes mellitus, hypertension,

prior cardiovascular disease, and untreated dyslipidemia), and calendar year of cohort entry
