# Supplementary material for: Traditional and HIV-specific risk factors for cardiovascular morbidity and mortality among HIV-infected adults in Brazil: a retrospective cohort study
Source: BMC Infect Dis. 2016 Aug 8;16:376. doi: 10.1186/s12879-016-1735-4 (PMC4977901; doi:10.1186/s12879-016-1735-4)
Supplement: Additional file 5: — Sensitivity analysis excluding VTEs, including a table that details the crude incidence rate ratios for demographic/clinical characteristics and cardiovascular risk factors (excluding VTEs), a table that details the final multivariate regression model with the exclusion of VTEs, and a table demonstrating the crude and adjusted incidence rate ratios for cardiovascular hospitalizations and deaths by exposure to specific ART agents (DOCX 199 kb) [file 12879_2016_1735_MOESM5_ESM.docx]

Sensitivity Analysis

A sensitivity analysis was conducted that excluded hospitalizations and death due to venous embolism and thrombosis, pulmonary embolism and portal vein thrombosis (abbreviated together as VTE) from the composite primary outcome.

The table below can be compared to supplemental table 3. The main differences when assessing composite hospitalizations and death are that race is no longer significantly associated with incident CVD-events once VTEs are excluded, and the effect size increases for age > 40 years, detectable viremia, history of hypertension. Additionally, with the exclusion of VTE, prior CVD (such as MI or stroke) is no longer significantly predictive of CVD-related hospitalizations.

**Supplementary Table 6. Crude Incidence Rate Ratios for Demographic/Clinical Characteristics and Cardiovascular Risk Factors Excluding VTE.**

|  | | **Composite CVD related end points NO VTE (n=79)** | | | **Composite CVD related end points VTE (n=30)** | | | **CVD-related Hospitalizations NO VTE (n=60)** | | | **CVD-related Hospitalizations VTE (n=29)** | | | **CVD-related death no VTE (n=32)** | | |
| --- | --- | --- | --- | --- | --- | --- | --- | --- | --- | --- | --- | --- | --- | --- | --- | --- |
|  |  | cIRR | 95% CI | p-value | cIRR | 95% CI | p-value | cIRR | 95% CI | p-value | cIRR | 95% CI | p-value | cIRR | 95% CI | p-value |
| **Participants characteristics** | |  |  |  |  |  |  |  |  |  |  |  |  |  | | |
| Age ≥ 40 years | | 3.21 | 2.02-5.11 | <0.001 | 1.11 | 0.54-2.31 | 0.78 | 2.88 | 1.70-4.87 | <0.001 | 1.18 | 0.56-2.46 | 0.67 | 3.64 | 1.72-7.68 | 0.001 |
| Male gender | | 1.18 | 0.74-1.88 | 0.49 | 2.01 | 0.86-4.69 | 0.11 | 1.43 | 0.82-2.48 | 0.21 | 1.93 | 0.82-4.51 | 0.13 | 1.01 | 0.50-2.07 | 0.97 |
| Non-white race | | 1.51 | 0.97-2.35 | 0.066 | 2.21 | 1.07-4.59 | 0.033 | 1.69 | 1.02-2.80 | 0.044 | 2.09 | 1.0-4.38 | 0.051 | 1.66 | 0.83-3.32 | 0.15 |
| ≤8 yrs education | | 1.93 | 1.21-3.09 | 0.006 | 1.08 | 0.53-2.22 | 0.83 | 2.21 | 1.27-3.85 | 0.005 | 1.02 | 0.49-2.11 | 0.97 | 2.41 | 1.12-5.21 | 0.025 |
| IDU exposure | | 1.54 | 0.48-4.87 | 0.47 |  |  |  | 2.05 | 0.64-6.54 | 0.23 |  |  |  | 1.26 | 0.17-9.20 | 0.82 |
| Heavy cocaine use | | 0.86 | 0.35-2.12 | 0.74 | 0.9 | 0.22-3.80 | 0.89 | 0.9 | 0.33-2.49 | 0.85 | 0.94 | 0.22-3.94 | 0.93 | 0.41 | 0.06-2.97 | 0.38 |
| Nadir CD4+ T -cell count ≤ 50 cells/mm3* | | 1.98 | 1.24-3.15 | 0.004 | 2.22 | 1.03-4.76 | 0.04 | 2.08 | 1.23-3.54 | 0.006 | 2.35 | 1.08-5.09 | 0.031 | 2.43 | 1.19-4.96 | 0.015 |
| Detectable viral load* | | 4.18 | 2.60-6.73 | <0.001 | 3.55 | 1.42-8.90 | 0.008 | 4.62 | 2.69-7.92 | <0.001 | 3.33 | 1.30-8.50 | 0.013 | 2.52 | 1.20-5.31 | 0.015 |
| Viremia copy-years, log10 copy x y/mL | | 0.91 | 0.70-1.18 | 0.48 | 1.57 | 0.94-2.61 | 0.084 | 0.99 | 0.73-1.34 | 0.95 | 1.51 | 0.90-2.53 | 0.12 | 0.73 | 0.49-1.09 | 0.13 |
| Time on ART/Years with known HIV | | 0.29 | 0.14-0.61 | 0.001 | 0.29 | 0.09-0.97 | 0.045 | 0.27 | 0.12-0.64 | 0.003 | 0.31 | 0.09-1.09 | 0.068 | 1.05 | 0.72-1.52 | 0.8 |
| **Cardiovascular risk factors** | | | | | | | | | | | | | | | | |
| History of diabetes mellitus | | 1.79 | 1.03-3.10 | 0.038 | 1.41 | 0.54-3.68 | 0.48 | 1.95 | 1.05-3.60 | 0.033 | 1.47 | 0.56-3.85 | 0.44 | 1.25 | 0.48-3.26 | 0.64 |
| History of hypertension | | 2.45 | 1.57-3.82 | <0.001 | 0.88 | 0.40-1.92 | 0.75 | 2.51 | 1.51-4.17 | <0.001 | 0.92 | 0.42-2.03 | 0.84 | 2.88 | 1.42-5.84 | 0.003 |
| History of dyslipidemia | | 1.00 | 0.64-1.56 | 0.99 | 0.39 | 0.19-0.82 | 0.014 | 0.89 | 0.53-1.48 | 0.64 | 0.36 | 0.17-0.77 | 0.008 | 1.12 | 0.55-2.29 | 0.76 |
| Prior CVD | | 5.00 | 2.81-8.91 | <0.001 | 1.66 | 0.40-6.96 | 0.49 | 3.07 | 1.39-6.74 | 0.005 | 1.72 | 0.41-7.23 | 0.46 | 10.47 | 5.05-21.72 | <0.001 |
| Ever smoked | | 2.62 | 1.45-4.75 | 0.001 | 1.21 | 0.55-2.63 | 0.64 | 1.87 | 1.01-3.45 | 0.06 | 1.15 | 0.52-2.52 | 0.73 |  |  |  |
| Weight tertiles* | Lowest third | 1.97 | 1.16-3.34 | 0.012 | 0.99 | 0.43-2.30 | 0.99 | 1.96 | 1.07-3.61 | 0.03 | 0.99 | 0.43-2.30 | 0.99 | 2.12 | 0.89-5.06 | 0.091 |
|  | Highest third | 0.8 | 0.43-1.48 | 0.48 | 0.62 | 0.25-1.51 | 0.29 | 0.84 | 0.42-1.68 | 0.62 | 0.54 | 0.21-1.37 | 0.2 | 1.05 | 0.41-2.74 | 0.91 |

With regard to the multivariate analysis for the composite outcome, with the exclusion of VTE, our final model would include: age ≥ 40 years, education, nadir CD4+ T cell count, detectable HIV-1 RNA, history of hypertension, prior CVD and smoking history (see table below).

**Supplementary Table 7. Multivariate Regression Model for Composite Outcome without VTEs**

|  | **Model 1- All Covariates** | | | **Model 2-Dropped IDU, heavy cocaine** | | | **Model 3- Additionally dropped weight** | | | **Model 4- Additionally dropped race** | | | **Model 5- Drop if p>0.1 (Final Model)** | | |
| --- | --- | --- | --- | --- | --- | --- | --- | --- | --- | --- | --- | --- | --- | --- | --- |
| **Covariates** | **IRR** | **95% CI** | **P-value** | **IRR** | **95% CI** | **P-value** | **IRR** | **95% CI** | **P-value** | **IRR** | **95% CI** | **P-value** | **IRR** | **95% CI** | **P-value** |
| Age ≥ 40 years | 2.87 | 1.73-4.74 | <0.001 | 2.9 | 1.76-4.77 | <0.001 | 2.88 | 1.75-4.75 | <0.001 | 2.88 | 1.75-4.74 | <0.001 | 2.93 | 1.78-4.81 | <0.001 |
| Male gender | 1.58 | 0.96-2.59 | 0.073 | 1.57 | 0.96-2.57 | 0.07 | 1.33 | 0.82-2.15 | 0.24 | 1.31 | 0.81-2.12 | 0.27 | - | - | - |
| Non-white race | 1.21 | 0.76-1.94 | 0.069 | 1.2 | 0.75-1.92 | 0.46 | 1.2 | 0.75-1.92 | 0.45 | - | - | - | - | - | - |
| ≤8 yrs education | 1.82 | 1.10-3.03 | 0.02 | 1.77 | 1.07-2.94 | 0.03 | 1.87 | 1.13-3.10 | 0.02 | 1.97 | 1.21-3.20 | 0.01 | 1.84 | 1.15-2.96 | 0.01 |
| IDU | 2.13 | 0.63-7.20 | 0.22 | - | - | - | - | - | - | - | - | - | - | - | - |
| Heavy cocaine use | 0.7 | 0.26-1.86 | 0.48 | - | - | - | - | - | - | - | - | - | - | - | - |
| Nadir CD4+ cell count ≤ 50 | 1.60 | 0.98-2.63 | 0.063 | 1.6 | 0.97-2.62 | 0.06 | 1.7 | 1.04-2.77 | 0.04 | 1.71 | 1.05-2.79 | 0.03 | 1.73 | 1.07-2.82 | 0.03 |
| Detectable HIV-1 RNA | 3.49 | 2.08-5.85 | <0.001 | 3.44 | 2.05-5.76 | <0.001 | 3.51 | 2.13-5.79 | <0.001 | 3.53 | 2.14-5.82 | <0.001 | 3.58 | 2.17-5.88 | <0.001 |
| % time with HIV on tx | 0.37 | 0.16-0.82 | 0.015 | 0.37 | 0.16-0.83 | 0.02 | 0.33 | 0.15-0.75 | 0.01 | 0.32 | 0.14-0.73 | 0.01 | 0.33 | 0.15-0.72 | 0.01 |
| Hx diabetes mellitus | 1.45 | 0.80-2.63 | 0.22 | 1.44 | 0.79-2.62 | 0.23 | 1.34 | 0.74-2.42 | 0.34 | 1.32 | 0.73-2.40 | 0.19 | - | - | - |
| Hx of hypertension | 1.99 | 1.22-3.23 | 0.006 | 1.97 | 1.21-3.20 | 0.01 | 1.76 | 1.08-2.86 | 0.02 | 1.78 | 1.09-2.88 | 0.02 | 1.77 | 1.10-2.85 | 0.02 |
| Hx of dyslipidemia | 0.91 | 0.55-1.49 | 0.7 | 0.91 | 0.55-1.49 | 0.71 | 0.86 | 0.53-1.42 | 0.56 | 0.85 | 0.52-1.40 | 0.53 | - | - | - |
| Hx prior CVD | 2.41 | 1.27-4.60 | 0.007 | 2.4 | 1.26-4.57 | 0.01 | 2.73 | 1.43-5.20 | 0.002 | 2.73 | 1.44-5.19 | 0.002 | 2.79 | 1.50-5.23 | 0.001 |
| Ever smoked | 1.67 | 0.91-3.07 | 0.1 | 1.68 | 0.91-3.09 | 0.09 | 1.77 | 0.96-3.24 | 0.07 | 1.78 | 0.97-3.26 | 0.06 | 1.83 | 1.00-3.34 | 0.05 |
| Weight tertile, 1st * | 1.78 | 1.03-3.09 | 0.04 | 1.78 | 1.02-3.08 | 0.04 | - | - | - | - | - | - | - | - | - |
| Weight tertile, 3rd * | 0.7 | 0.38-1.200 | 0.26 | 0.69 | 0.37-1.28 | 0.24 | - | - | - | - | - | - | - | - | - |
| Calendar year cohort entry | 0.99 | 0.95-1.03 | 0.58 | 0.98 | 0.95-1.03 | 0.46 | 0.99 | 0.95-1.03 | 0.65 | 0.99 | 0.95-1.03 | 0.63 | - | - | - |

When specific ART exposure is added to the model, the results are similar to our initial analysis (Table 5 of the manuscript) with few exceptions (highlighted below).

| **Supplementary Table 8: Crude and Adjusted* Incidence Rate Ratios for Cardiovascular Events by Exposure to Specific Antiretroviral Drugs** | | | | | | | | | | | | |
| --- | --- | --- | --- | --- | --- | --- | --- | --- | --- | --- | --- | --- |
|  |  |  |  |  |  |  |  |  |  |  |  |  |
|  | **Composite CV endpoint** | | | | | | | | | | | |
|  | **Cumulative Exposure no-VTE** | | | | | | **Recent Exposure** | | | | | |
|  | cIRR | 95% CI | P | aIRR | 95% CI | P | cIRR | 95% CI | P | aIRR | 95% CI | P |
| **ART** | 0.86 | 0.82-0.91 | <0.001 | 0.8 | 0.75-0.85 | <0.001 | - | - | - | - | - | - |
| **NRTIs** | 0.86 | 0.82-0.91 | <0.001 | 0.8 | 0.75-0.85 | <0.001 | - | - | - | - | - | - |
| Lamivudine | 0.81 | 0.76-0.86 | <0.001 | 0.77 | 0.72-0.83 | <0.001 | 0.49 | 0.24-0.98 | 0.04 | 0.59 | 0.29-1.21 | 0.15 |
| Zidovudine | 0.87 | 0.82-0.93 | <0.001 | 0.83 | 0.77-0.89 | <0.001 | 1.08 | 0.68-1.70 | 0.75 | 1.06 | 0.67-1.69 | 0.80 |
| Tenofovir | 0.65 | 0.54-0.78 | <0.001 | 0.62 | 0.51-0.75 | <0.001 | 0.38 | 0.23-0.62 | <0.001 | 0.34 | 0.21-0.57 | <0.001 |
| Stavudine | 0.99 | 0.91-1.08 | 0.9 | 0.96 | 0.88-1.06 | 0.44 | 6.54 | 3.89-10.98 | <0.001 | 5.53 | 3.10-9.85 | <0.001 |
| Didanosine | 0.95 | 0.85-1.06 | 0.34 | 0.88 | 0.78-1.01 | 0.06 | 0.85 | 0.27-2.69 | 0.78 | 0.86 | 0.27-2.76 | 0.80 |
| Emtricitabine | 0.88 | 0.56-1.38 | 0.57 | 1.07 | 0.66-1.72 | 0.79 | 3.20 | 1.01-10.17 | 0.05 | 5.46 | 1.65-18.1 | 0.01 |
| Abacavir | 0.72 | 0.42-1.23 | 0.23 | 0.66 | 0.35-1.24 | 0.19 | 0.67 | 0.09-4.80 | 0.69 | 0.38 | 0.05-2.80 | 0.35 |
| Zalcitabine | 1.01 | 0.61-1.67 | 0.98 | 0.88 | 0.52-1.51 | 0.65 | - | - | - | - | - | - |
| **NNRTIs** | 0.78 | 0.70-0.86 | <0.001 | 0.79 | 0.71-0.88 | <0.001 | 1.05 | 0.67-1.65 | 0.83 | 1.60 | 0.99-2.58 | 0.05 |
| Efavirenz | 0.78 | 0.69-0.88 | <0.001 | 0.8 | 0.70-0.90 | <0.001 | 0.81 | 0.50-1.30 | 0.38 | 1.24 | 0.75-2.05 | 0.41 |
| Nevirapine | 0.92 | 0.79-1.08 | 0.32 | 0.89 | 0.75-1.07 | 0.22 | 3.03 | 1.39-6.59 | 0.01 | 4.22 | 1.87-9.51 | 0.001 |
| **PIs** | 0.92 | 0.87-0.97 | 0.004 | 0.87 | 0.82-0.93 | <0.001 | 1.23 | 0.76-1.98 | 0.40 | 0.70 | 0.42-1.17 | 0.18 |
| **PIs (without high dose RTV)** | 0.92 | 0.87-0.97 | 0.004 | 0.87 | 0.81-0.92 | <0.001 | 1.16 | 0.72-1.86 | 0.54 | 0.65 | 0.39-1.09 | 0.11 |
| Ritonavir | 0.93 | 0.79-1.10 | 0.38 | 0.87 | 0.72-1.05 | 0.15 | 4.39 | 2.02-9.54 | <0.001 | 2.61 | 1.14-5.97 | 0.02 |
| Lopinavir/Ritonavir | 0.88 | 0.78-1.00 | 0.06 | 0.83 | 0.72-0.96 | 0.01 | 1.15 | 0.69-1.90 | 0.59 | 0.79 | 0.47-1.32 | 0.37 |
| Atazanavir + Ritonavir | 0.56 | 0.38-0.81 | 0.002 | 0.55 | 0.37-0.82 | 0.003 | 0.24 | 0.10-0.60 | 0.002 | 0.25 | 0.10-0.63 | 0.003 |
| Nelfinavir without booster | 0.92 | 0.79-1.08 | 0.31 | 0.87 | 0.74-1.06 | 0.18 | 7.49 | 3.44-16.29 | <0.001 | 5.26 | 2.29-12.11 | <0.001 |
| Indinavir without booster | 1.08 | 0.98-1.20 | 0.13 | 1.02 | 0.91-1.14 | 0.76 | 14.51 | 6.97-30.19 | <0.001 | 10.85 | 4.65-25.28 | <0.001 |
| Saquinavir + Ritonavir | 0.43 | 0.05-3.63 | 0.44 | 0.28 | 0.02-3.77 | 0.34 | - | - | - | - | - | - |
| Saquinavir without booster | 0.94 | 0.74-1.19 | 0.35 | 0.88 | 0.68-1.15 | 0.35 | 2.7 | 0.66-11.02 | 0.17 | 1.24 | 0.29-5.37 | 0.77 |
| Darunavir + Ritonavir | 0.66 | 0.40-1.08 | 0.1 | 0.61 | 0.36-1.02 | 0.06 | 0.59 | 0.22-1.61 | 0.30 | 0.46 | 0.17-1.29 | 0.14 |
| Amprenavir + Ritonavir | 1.75 | 0.94-3.24 | 0.08 | 1.59 | 0.78-3.24 | 0.2 | - | - | - | - | - | - |
| Amprenavir without booster | 0.96 | 0.51-1.83 | 0.91 | 0.83 | 0.38-1.80 | 0.64 | 13.34 | 1.86-95.96 | 0.01 | 6.15 | 0.81-46.51 | 0.08 |
| **Integrase Inhibitors** | 0.93 | 0.61-1.42 | 0.74 | 0.91 | 0.60-1.37 | 0.65 | 0.87 | 0.32-2.39 | 0.79 | 0.84 | 0.30-2.34 | 0.74 |
| **Fusion Inhibitors** | 0.87 | 0.51-1.47 | 0.59 | 0.79 | 0.46-1.37 | 0.41 | 2.65 | 0.65-10.78 | 0.18 | 1.18 | 0.28-4.99 | 0.82 |

* Final model was adjusted for age ≥ 40 years, education, nadir CD4+ T cell count, detectable HIV-1 RNA, history of hypertension, prior CVD, smoking history, and calendar year (the ratio of time on ART was removed due to collinearity)
